# Multimodal imaging analysis in silver fir reveals coordination in cellulose and lignin deposition

**DOI:** 10.1093/plphys/kiae203

**Published:** 2024-04-09

**Authors:** Gonzalo Pérez-de-Lis, Béatrice Richard, Fabienne Quilès, Aurélie Deveau, Ignatius-Kristia Adikurnia, Cyrille B K Rathgeber

**Affiliations:** BIOAPLIC, Departamento de Botánica, Universidade de Santiago de Compostela, EPSE, Campus Terra, 27002 Lugo, Spain; Université de Lorraine, AgroParisTech, INRAE, SILVA, F-54000 Nancy, France; Université de Lorraine, AgroParisTech, INRAE, SILVA, F-54000 Nancy, France; Université de Lorraine, CNRS, LCPME, F-54000 Nancy, France; Université de Lorraine, INRAE, IAM, F-54000 Nancy, France; Université de Lorraine, AgroParisTech, INRAE, SILVA, F-54000 Nancy, France; Université de Lorraine, AgroParisTech, INRAE, SILVA, F-54000 Nancy, France

## Abstract

Despite lignin being a key component of wood, the dynamics of tracheid lignification are generally overlooked in xylogenesis studies, which hampers our understanding of environmental drivers and blurs the interpretation of isotopic and anatomical signals stored in tree rings. Here, we analyzed cell wall formation in silver fir (*Abies alba* Mill.) tracheids to determine if cell wall lignification lags behind secondary wall deposition. For this purpose, we applied a multimodal imaging approach combining transmitted light microscopy (TLM), confocal laser scanning microscopy (CLSM), and confocal Raman microspectroscopy (RMS) on anatomical sections of wood microcores collected in northeast France on 11 dates during the 2010 growing season. Wood autofluorescence after laser excitation at 405 and 488 nm associated with the RMS scattering of lignin and cellulose, respectively, which allowed identification of lignifying cells (cells showing lignified and nonlignified wall fractions at the same time) in CLSM images. The number of lignifying cells in CLSM images mirrored the number of wall-thickening birefringent cells in polarized TLM images, revealing highly synchronized kinetics for wall thickening and lignification (similar timings and durations at the cell level). CLSM images and RMS chemical maps revealed a substantial incorporation of lignin into the wall at early stages of secondary wall deposition. Our results show that most of the cellulose and lignin contained in the cell wall undergo concurrent periods of deposition. This suggests a strong synchronization between cellulose and lignin-related features in conifer tree-ring records, as they originated over highly overlapped time frames.

## Teaser

Multiple imaging techniques applied to the analysis of xylogenesis in silver fir reveal analogous deposition timeframes for cellulose and lignin at both cellular and tissue levels.

## Introduction

Lignin is the second most abundant group of natural polymers on Earth after cellulose and represents up to 30% of the organic carbon in the biosphere (Boerjan et al. 2003). This implies that a substantial part of the carbon taken by the tree leaves is sequestered in the form of lignin. It also plays a major role in the physiology of vascular plants, conferring compressive strength and water impermeability to supporting and conducting xylem cells, which accounts for their superior mechanical and hydraulic resistance (Vanholme et al. 2010; Voelker et al. 2011; Pereira et al. 2018). Lignin also contributes to woody plants’ defense against pathogens, increasing their resistance to degradation by microbial attack (Ranade et al. 2022). On the other hand, multiple industrial uses of lignocellulosic materials highlight the major economic importance of lignin biosynthesis, including the development of materials such as wood–polymer composites, lignin-based adhesives, nanoparticles, or carbon fibers among others (Norgren and Edlund 2014; Leng et al. 2022). However, although tree-ring research has contributed to shedding light on the environmental constraints of lignification (Piermattei et al. 2020), the underpinnings of the interaction between xylem developmental factors and climate are still far from being understood.

Lignin is a phenolic heteropolymer synthesized during xylogenesis, which is characterized by the following phases: cell division, cell enlargement, wall thickening (i.e. secondary cell wall [SCW] deposition and cell wall lignification), and programmed cell death (Rathgeber et al. 2016). After division, mother xylem cells experience irreversible lumen enlargement through primary cell wall (PCW) extension, after which the S1, S2, and S3 layers of the SCW and the warty layer are formed. Lignification begins in initiation sites located in cell corner (CC) and middle lamella (interface between 2 adjacent tracheids), spreading later inward into the SCW (Donaldson 2001; Schmitt et al. 2003) and concluding after cell death (Pesquet et al. 2013; Meents et al. 2018). During lignification, interlamellar voids left by cellulose microfibrils are filled by lignin, which forms chemical bonds with hemicelluloses. Therein, monolignols previously synthetized in the cell lumen are incorporated into polymers through the action of peroxidases (Boerjan et al. 2003; Vanholme et al. 2010; Tobimatsu et al. 2013). SCW deposition is known to occur after cell enlargement, resulting in a lag effect between growth in size and biomass in tree stems (Andrianantenaina et al. 2019). Likewise, previous studies converge on the idea that lignification follows SCW deposition. However, the precise time lag between these 2 phases at the cell and tissue levels remains largely unknown. Some authors argued that the SCW would be largely or even totally developed before lignification (Donaldson 1991; Fukushima and Terashima 1991; Donaldson 2001), while more recent studies suggest that lignification may initiate soon after the polysaccharide matrix starts to be deposited (Joseleau and Ruel 2006; Meents et al. 2018). This knowledge gap hampers an accurate consideration of the contribution of cellulose and lignin to the intraannual dynamics of stem biomass growth.

**Figure 1.**
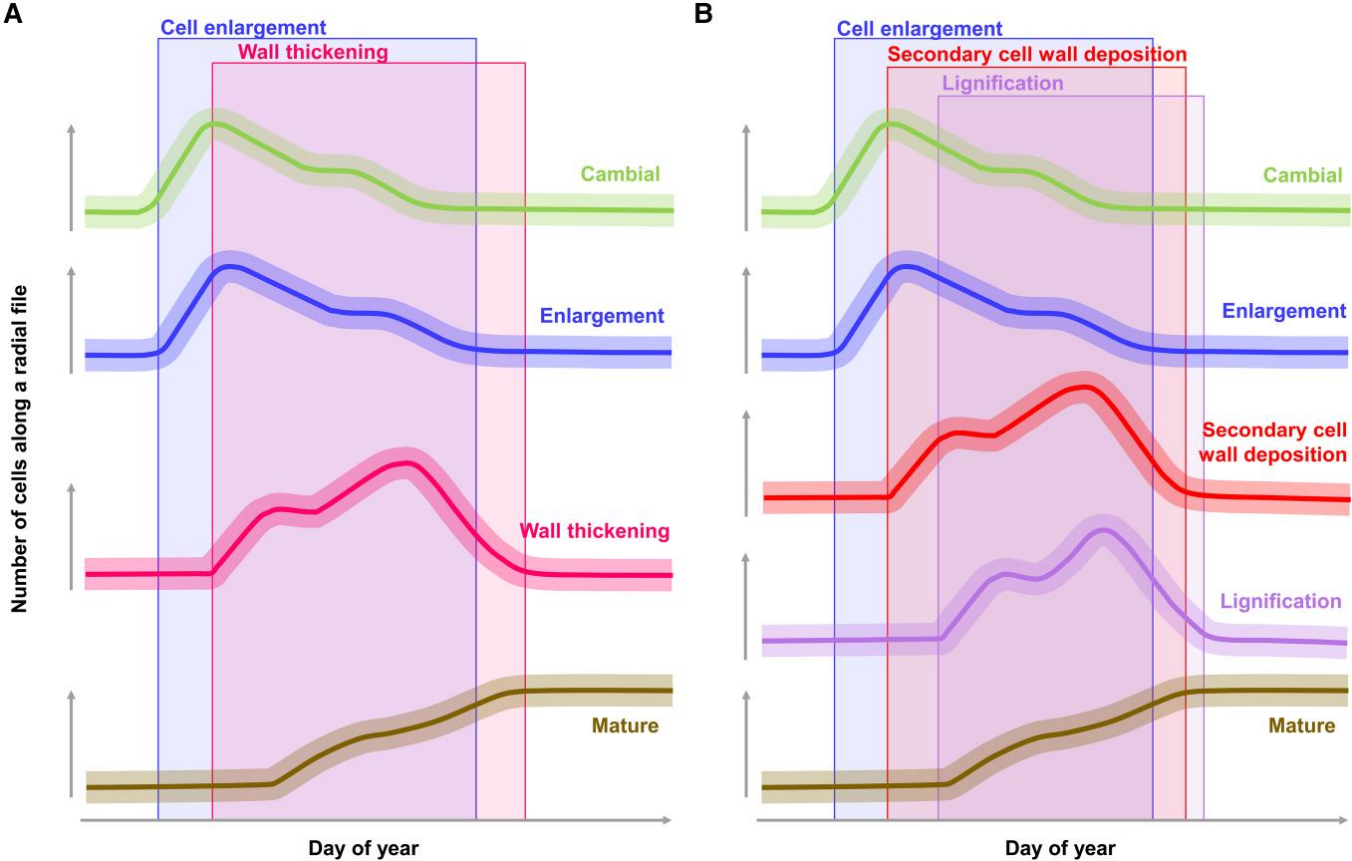
Theoretical scheme of intraannual dynamics of xylogenesis in temperate conifers. Curves provided in the literature **A)** based on the variation in the number of cells counted across the 4 main zones (cambial, enlarging, wall thickening, and mature) and **B)** considering SCW deposition and cell wall lignification as 2 separated differentiation processes. Background boxes represent theoretical time periods of the corresponding phenophases.

Examination of wood microcores repeatedly collected throughout the growing season by using transmitted light microscopy (TLM) has contributed to a mechanistic understanding of xylogenesis processes and their respective environmental drivers (Cuny et al. 2019). In gymnosperms, the assessment of intraannual dynamics of wood formation has been possible thanks to the application of a data-driven modeling approach on differentiating and mature cell counts obtained along radial files from transverse microsections (Cuny et al. 2013). Changes in the radial number of tracheids across mature, wall-thickening, enlarging, and cambial zones over time can be used to estimate the time spent by tracheids in consecutive differentiation phases (Fig. 1). However, cells undergoing SCW deposition and lignification have often been included within the same wall-thickening zone, using (i) polarized light to flag the onset of SCW deposition thanks to birefringence of cellulose microfibrils and (ii) different dye combinations to mark the end of lignification (Rathgeber et al. 2016). As a result, intraannual dynamics of SCW deposition and lignification have rarely been compared, obscuring the interpretation of inter- and intraring series of related traits (Pérez-de-Lis et al. 2022). This could be in part attributed to difficulty in detecting early lignification stages due to limited contrast and resolution achieved in TLM images (Bond et al. 2008), which advocates for the use of more powerful imaging tools to study lignification.

Advanced imaging techniques (e.g. transmission electron microscopy) have been used to render a precise evaluation of SCW deposition and lignification in both conifers (Schmitt et al. 2003; Gričar et al. 2005) and angiosperms (Prislan et al. 2009). Xylem composition has also been analyzed by leveraging on natural wood fluorescence (Donaldson 2013, 2020), employing techniques like ultraviolet microspectrophotometry (e.g. Schmitt et al. 2003). Confocal laser scanning microscopy (CLSM) is another fluorescence-based tool used to assess cell wall ultrastructure (Grünwald et al. 2002; Donaldson et al. 2010; Kitin et al. 2020), as well as to monitor lignification (Dickson et al. 2017; Nanayakkara et al. 2019; Balzano et al. 2021). This has been possible because lignin polyphenolic aromatic rings produce fluorescent signals following excitation by ultraviolet and visible lights. Yet, wood autofluorescence does not only depend on lignin, as cellulose and hemicellulose unsaturated glycosidic bonds also emit fluorescence (Ding et al. 2020). Thus, some authors have proposed to label lignin with fluorescent dyes and antibodies to visualize lignin-rich tissues (Kitin et al. 2003; Joseleau and Ruel 2006; Bond et al. 2008; Tobimatsu et al. 2013; Kitin et al. 2020; Balzano et al. 2021). On the other hand, analytical tools such as confocal Raman microspectroscopy (RMS) have been used to study the chemistry of cell wall, as it renders spatial information on the distribution of lignin and other cell wall constituents (Agarwal 1999; Gierlinger et al. 2012; Guillon et al. 2022; Leng et al. 2022). Therefore, combining TLM-derived observations with spatial information provided by CLSM and RMS could bring valuable information on the dynamics of lignification.

This study aimed to analyze SCW deposition and lignification in silver fir (*Abies alba* Mill.) tracheids through a multimodal imaging approach. First, we analyzed the presence of lignin on transverse wood microsections by using RMS and CLSM images. To assess the offset between wall thickening (i.e. SCW deposition and cell wall lignification) and lignification, we characterized their respective dynamics (Fig. 1) through radial cell counts obtained from TLM and CLSM images. Three main hypotheses were tested: (i) at the tissue level, lignin concentrations decrease from mature xylem toward the cambial zone according to the degree of cell differentiation; (ii) at the cell level, SCW deposition starts earlier than cell wall lignification; and (iii) timings and durations of wall thickening and lignification respectively obtained through TLM and CLSM reflect the delay between SCW deposition and cell wall lignification.

## Results

### Characteristic Raman and autofluorescence signals in mature xylem and the vascular cambium

**Figure 2.**
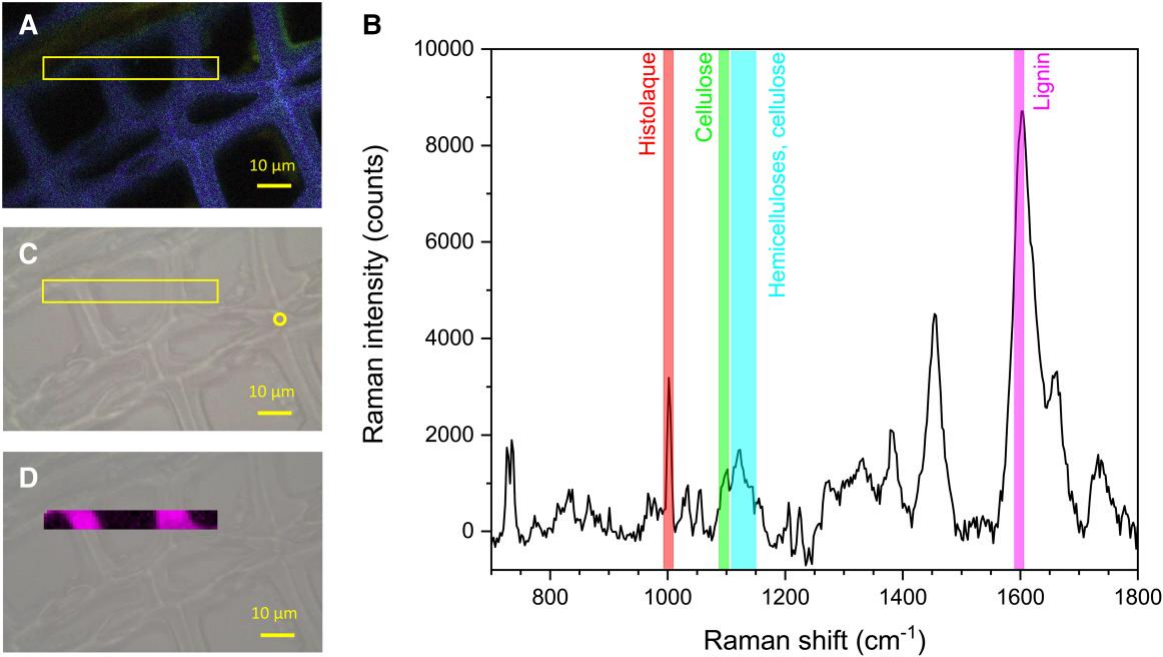
RMS exploration of mature xylem. **A)** Autofluorescence image showing the spectral emission (415 to 691 nm) following a single 405 nm excitation on 32 colored channels using the lambda mode spectra of CLSM. **B)** Raman spectrum recorded on the middle lamella between 2 mature tracheids in the area delimited by a circle in the TLM image **C)**. **D)** Raman chemical image (box in **A** and **C**) of the cell wall area based on the lignin specific band at 1,599 cm^−1^ (integrated intensities between 1,592 and 1,604 cm^−1^). Magnification: 50× objective for the spectra and 80× objective for the hyperspectral map.

**Figure 3.**
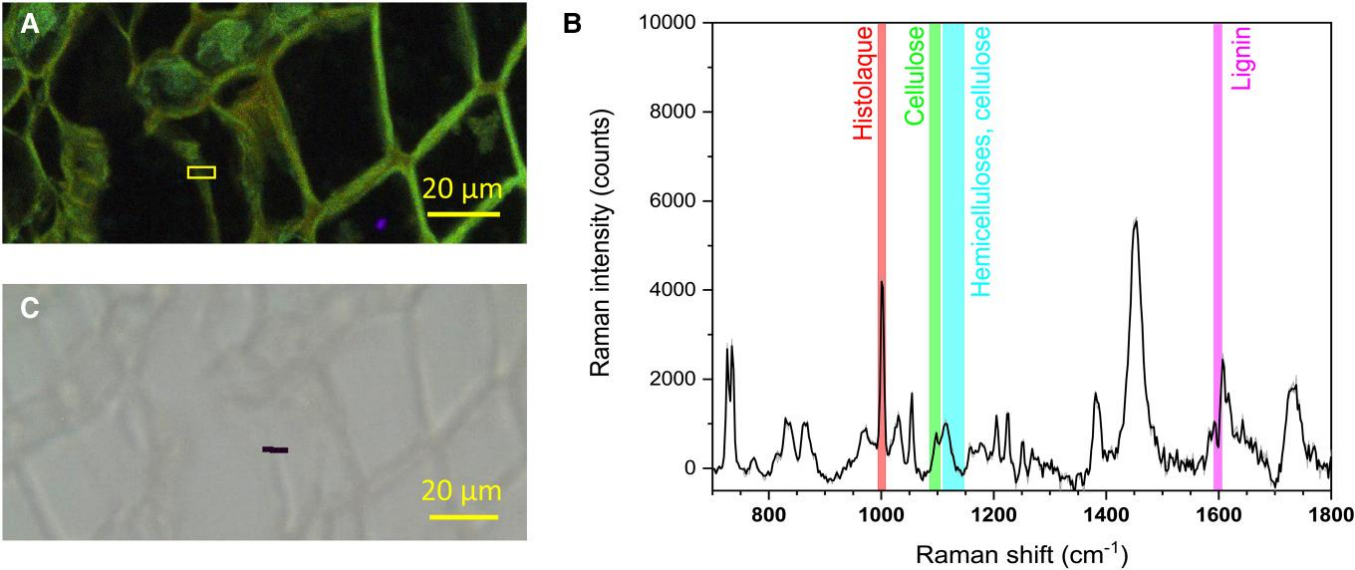
RMS exploration of the cambial zone. **A)** Autofluorescence image showing the spectral emission (415 to 691 nm) following a single 405 nm excitation on 32 colored channels using the lambda mode spectra of CLSM. **B)** Average Raman spectrum from 15 individual spectra recorded within the region of interest (box in **A**) and **C)** TLM image including the Raman chemical image on the lignin-specific band at 1,599 cm^−1^ (integrated intensities between 1,592 and 1,604 cm^−1^). Magnification: 50× objective.

The RMS spectrum recorded at the compound middle lamella (CML) of a mature tracheid (Fig. 2) showed the spectral features of the 3 structural polymers of xylem (cellulose, hemicelluloses, and lignin). It is also worth noting the contribution to the spectrum of Histolaque proteins through the Phe breathing mode of the aromatic ring at 1,003 cm^−1^. The lignin band at 1,599 cm^−1^ was the most prominent of the spectrum (Fig. 2), exhibiting an integrated intensity 5-fold higher than the one for cellulose. Moreover, the RMS chemical image showed that lignin was well distributed across the mature tracheid wall (Fig. 2), with celluloses, hemicelluloses, and background scattering related to Histolaque proteins consistently showing a more limited intensity (Supplementary Fig. S1). Conversely, the RMS spectrum obtained from the thin tangential wall of a cambial cell lacked the spectroscopic signature of lignin at 1,599 cm^−1^ (Fig. 3), whereas the other wood components (i.e. cellulose and hemicelluloses) were detected conjointly to Histolaque proteins.

**Figure 4.**
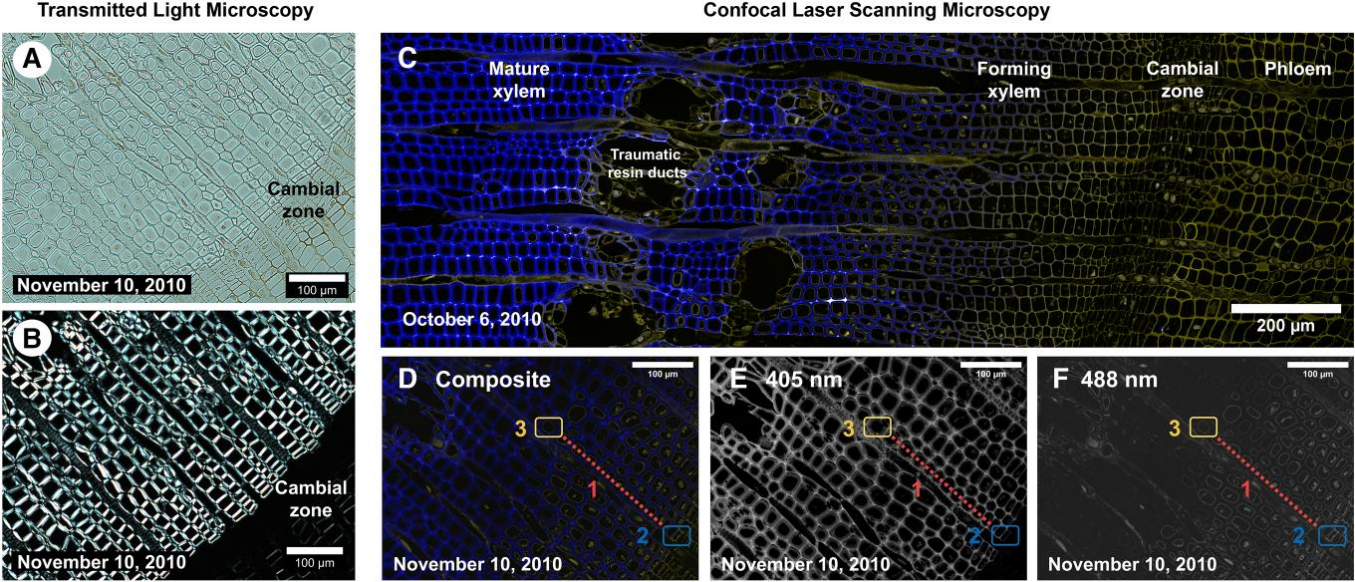
TLM and CLSM images of transverse wood microsections. Transmitted light image of unstained microsection showing xylem and cambial zone under white light **A)** and polarized light **B)**. **C)** Composite autofluorescence image of xylem, vascular cambium, and phloem showing the emission signal of different fluorophores excited simultaneously at 405 and 488 nm after applying linear unmixing spectra. **D)** Composite image of differentiating xylem and corresponding gray-scale images after splitting the signals excited at 405 nm **E)** and 488 nm **F)**. Dotted line 1 in **D to F)** highlight the range of lignifying cells along 1 radial file, defined by the relative dominance of the fluorophores excited at 405 and 488 nm. Boxe 2 in **D to F)** highlight the youngest lignifying cell, with fluorescence excited at 405 nm being dominant in the middle lamella. Box 3 in **D to F)** highlight the oldest lignifying cell, with fluorescence excited at 488 nm being dominant in the S3 layer.

The autofluorescence emitted by the xylem was mainly due to cell walls, followed by the protoplast of living cells (Fig. 4). A strong fluorescent emission occurred in mature tracheids at 442 to 513 nm after excitation with the 405 nm laser beam (represented in blue in composite CLSM images, Fig. 4, Supplementary Fig. S2), while that in cambial cells was very weak and lagged up to 504 to 575 nm. This emission was present through the entire cell wall, allowing easy recognition of earlywood and latewood tracheids (Supplementary Fig. S2). A weak fluorescent emission signal was captured in both mature tracheids (513 to 575 nm) and cambial and phloem cells (531 to 600 nm) for the 488 nm laser beam (represented in yellow in CLSM images, Fig. 4, Supplementary Fig. S2). Thus, while most of the mature xylem autofluorescence emission was excited at 405 nm, the autofluorescence of phloem and cambial cell walls was emitted only after excitation by the 488 nm laser beam. Microsections occasionally showed traumatic resin ducts and epithelial cells, which fluorescence emission occurred exclusively upon excitation at 488 nm (Fig. 4). Histolaque proteins did not exhibit fluorescence emission after excitation by 405 or 488 nm laser beams.

### Cell-level changes in Raman and autofluorescence signals during xylem differentiation

**Figure 5.**
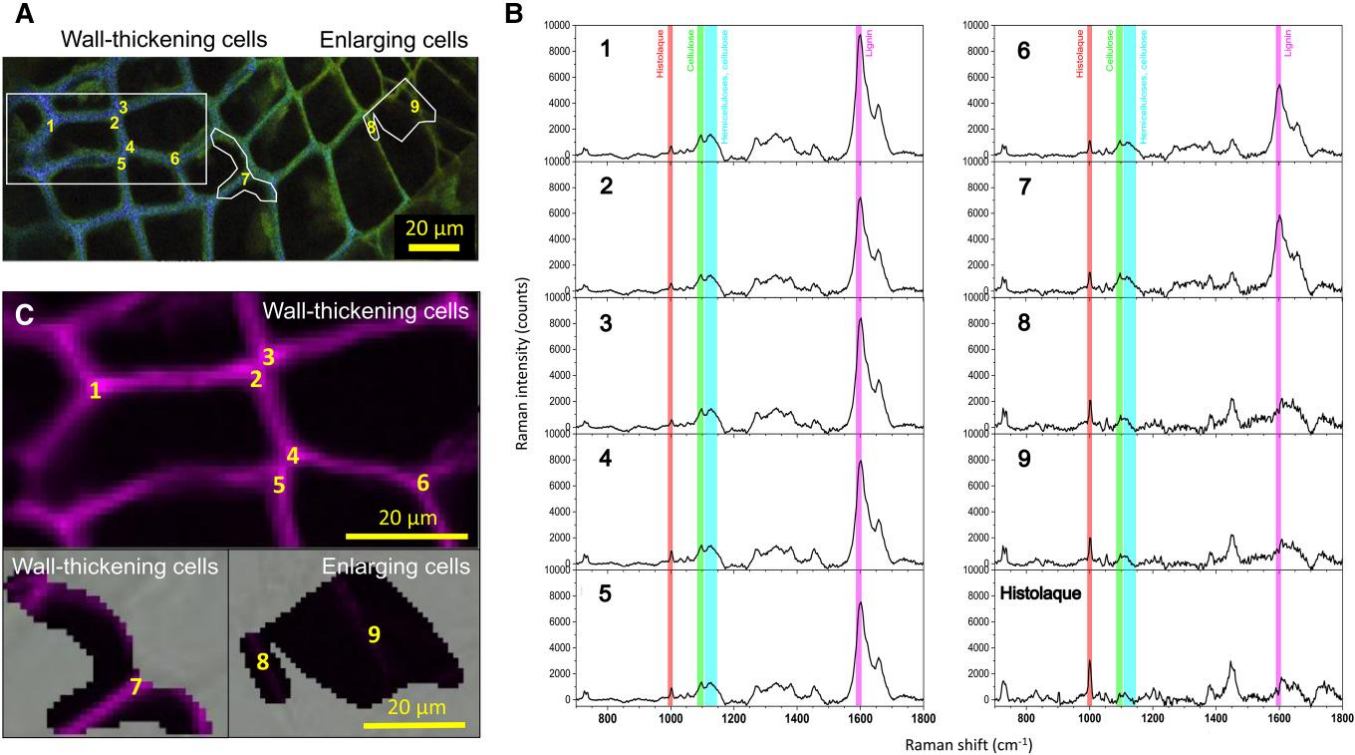
RMS exploration of differentiating xylem. **A)** Autofluorescence image showing the spectral emission (415 to 691 nm) following a single 405 nm excitation on 32 colored channels using the lambda mode spectra of CLSM. Nine locations of spectra recording along 3 regions of interest: 2 including wall-thickening tracheids on the left (locations 1 to 7) and 1 including thin-walled enlarging tracheids on the right (locations 8 and 9). **B)** Average spectrum from 4 individual spectra recorded in locations 1 to 9, each averaged over an area of 4 pixels (pixel = 1 *µ*m × 1 *µ*m). Pure Histolaque spectrum is added for comparison. **C)** Raman chemical images are based on the lignin-specific band at 1,599 cm^−1^ (integrated intensities between 1,592 and 1,604 cm^−1^). Magnification: 50× objective.

**Figure 6.**
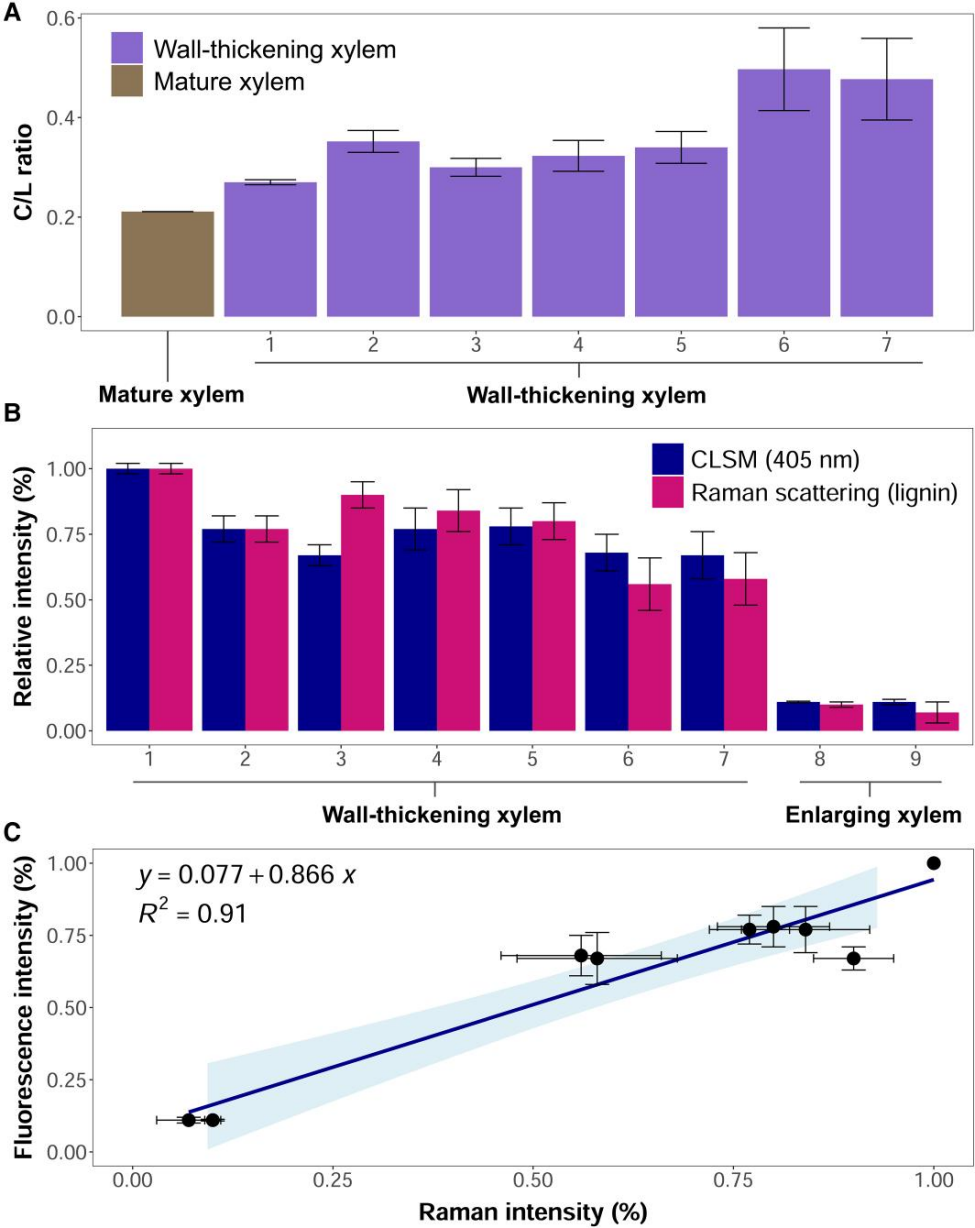
Relative lignin concentrations across differentiating xylem. **A)** C/L ratios calculated from the relative intensity of the RMS scattering for lignin and cellulose bands in mature (location in Fig. 3B) and wall-thickening xylem (locations 1 to 7 in Fig. 5A). **B)** Comparison of the relative intensity of the Raman scattering for the lignin band and autofluorescence signal related to lignin (excited at 405 nm) across differentiating xylem (locations 1 to 9 in Fig. 5A). **C)** Linear regression showing high correspondence between Raman and autofluorescence signals. RMS imaging of lignin at 1,592 to 1,604 cm^−1^ and cellulose at 1085 to 1,105 cm^−1^ (excitation laser: 532 nm). CLSM mapping of autofluorescence at 468 nm (excitation laser: 405 nm). Error bars represent the Sd (sample size: 4 pixels).

In developing xylem, RMS hyperspectral maps were recorded in the CC and CML over 9 different locations within the wall-thickening (1 to 7) and enlarging (8 to 9) zones (Fig. 5). The RMS spectra from enlarging tracheids (8 to 9, Fig. 5) greatly differed from those yielded in wall-thickening ones (1 to 7, Fig. 5). Whereas the tangential walls in 8 and 9 lacked the spectroscopy signature for lignin at 1,599 cm^−1^, an intense signal was observed in the RMS spectra in 1 to 7. The greatest change in the cellulose-to-lignin (C/L) ratio (indicating intense lignification) within the wall-thickening zone was recorded between locations 5 and 6 (Fig. 6, Supplementary Fig. S3), while most of the variation (48 out of 93%) in the relative intensity of the RMS scattering for the lignin band across the entire differentiation zone occurred between locations 7 and 8 (Fig. 6). These results indicated that lignin was deposited in the middle lamella well before the end of SCW deposition. Indeed, the spectra measured in locations 1 to 5 closely resembled those of mature xylem (compare Fig. 2 with Fig. 5). Moreover, the limited change in the C/L ratio (from 0.21 to 0.34) recorded between mature xylem and locations 1 to 5 pointed out that middle lamella hardly lignified after cell death or even during late stages of SCW deposition (Fig. 6). Changes in the relative signal intensity within the enlarging zone (locations 8 and 9) were limited to a more prominent signal for the cellulose spectral band in location 8 (Supplementary Fig. S3), indicating an increased cellulose content in the PCW.

### Changes in lignin deposition across cell wall layers

The relative signal intensity of the RMS spectra for the lignin band and the autofluorescence emission excited at 405 nm recorded at the same locations within the differentiation zone showed remarkably similar values (Fig. 6). This was confirmed by a linear regression between the 2 signals (*R*^2^ = 0.91, Fig. 6), pointing out a high correspondence between the autofluorescence emission excited at 405 nm (blue color in Fig. 4) and lignin content. Such correspondence enabled a detailed analysis of lignin concentrations across cell wall layers based on highly resolved CLSM images. Thereby, CC and CML were found to be more lignified than SCW, except for the S3 layer, which was rich in lignin (Fig. 4, Supplementary Fig. S2). Indeed, the S2 layer could be easily distinguished from the S3 and CML thanks to its lower autofluorescence signal intensity (Supplementary Fig. S2), being much thicker in latewood than in earlywood tracheids. More interestingly, lignin appeared to be present in middle lamella of still thin-walled tracheids (especially in CC), confirming that lignification was underway at very initial stages of SCW formation (Fig. 4). Although lignin was also present in the SCW in older wall-thickening cells, a lignin-deficient layer lined the inner cell wall boundary, revealing a temporal offset between cellulose microfibril deposition and lignin impregnation within the SCW (Fig. 4, but see Supplementary Fig. S2 for higher detail). The width of the lignin-deficient layer appeared not to vary regardless of the considerable change in wall thickness across differentiating tracheids.

### Intraannual dynamics of wall thickening and lignification at cell and tissue levels

**Figure 7.**
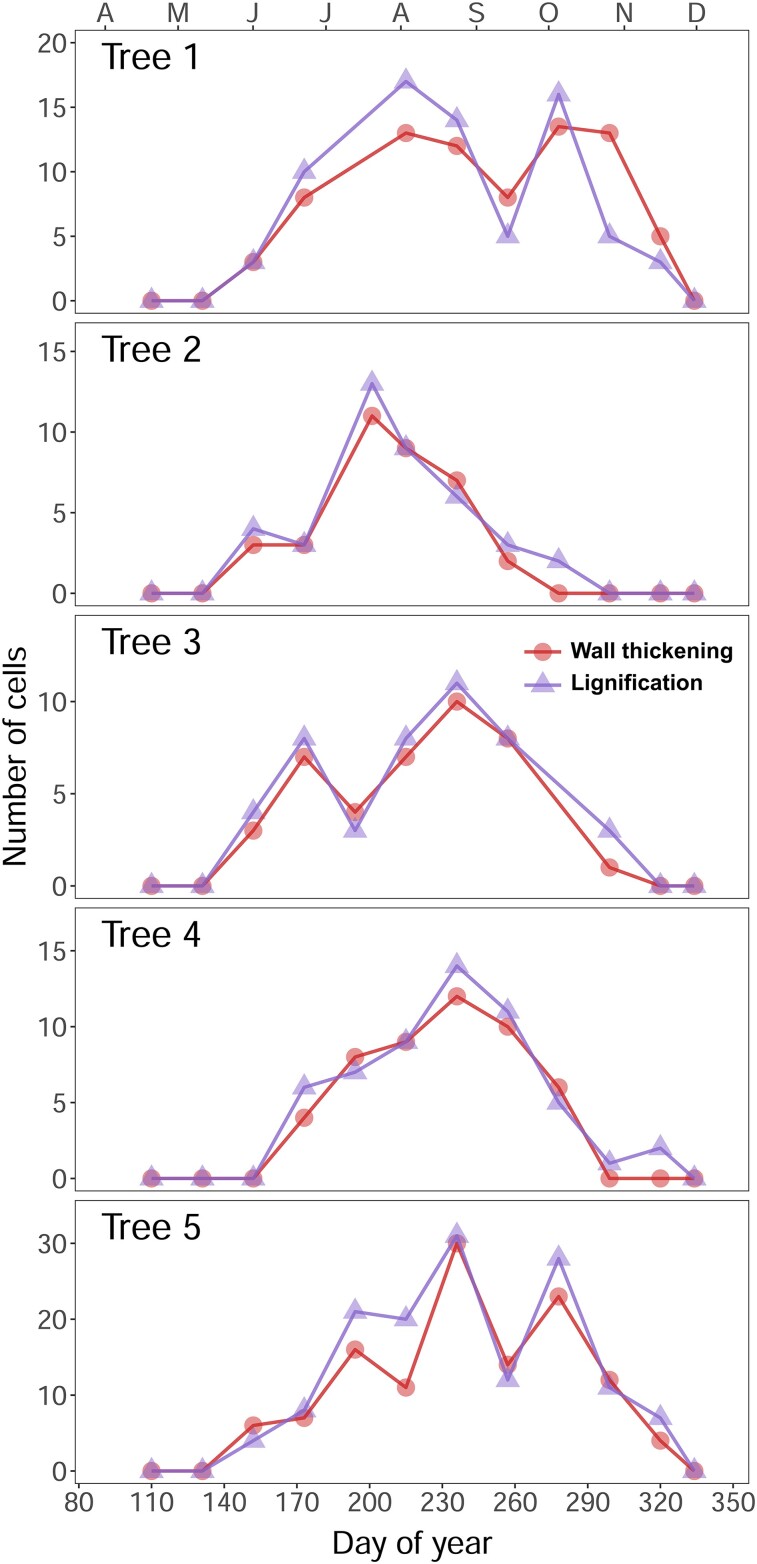
Intraannual variation in the number of wall-thickening and lignifying tracheids. Wall-thickening tracheids (circles) were measured in 5 trees under a transmitted light microscope, and lignifying tracheids (triangles) were obtained after processing autofluorescence images. Symbols (dots and triangles) represent averaged cell counts of 3 radial files for each date and phase.

**Figure 8.**
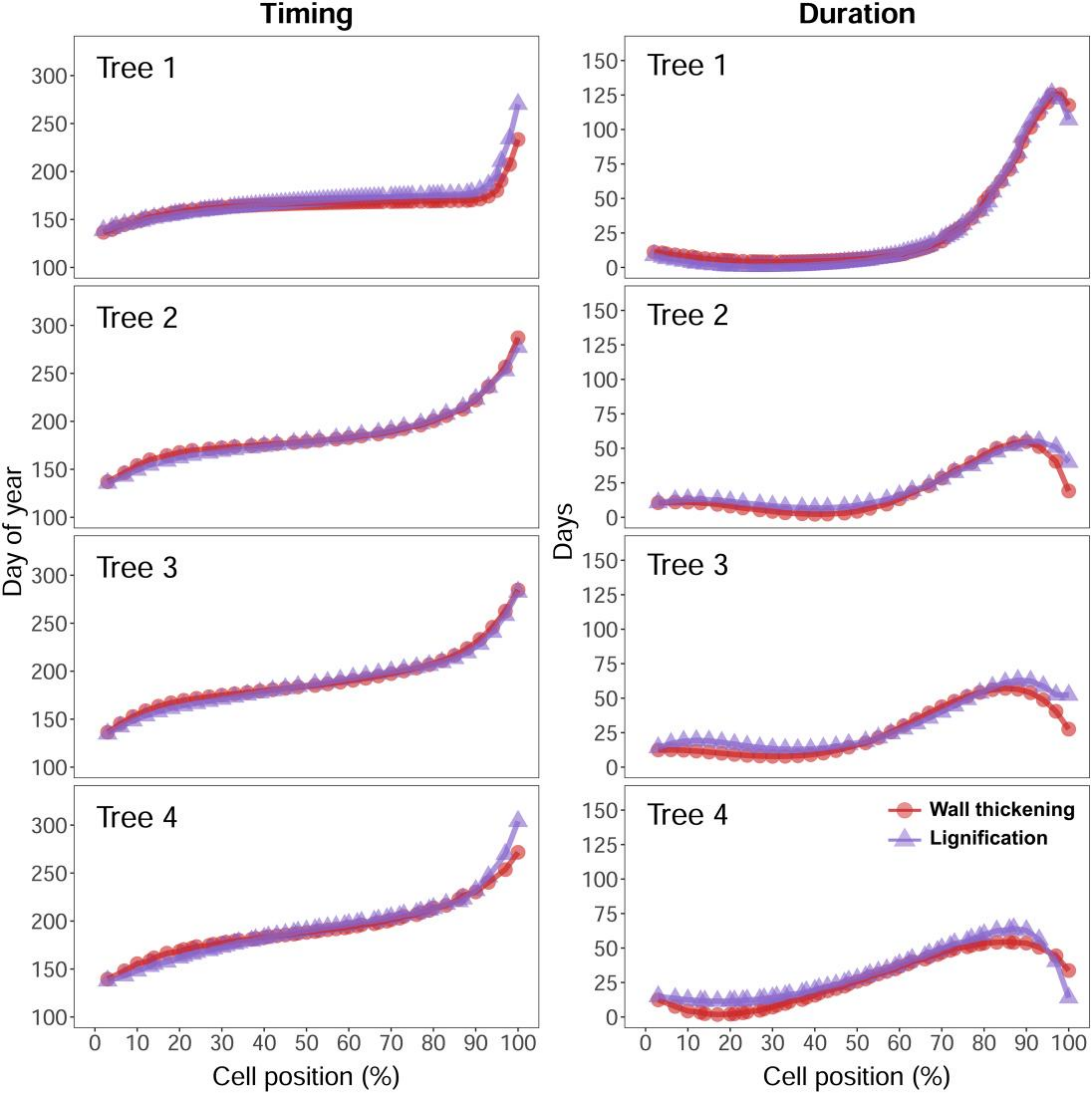
Cell kinetics of wall thickening and lignification. Cell-level timing (left) and duration (right) of wall thickening (circles) and lignification (triangles) were computed in 4 trees as the time lags between the model-predicted curves from either wall thickening or lignifying cell counts and mature cell counts. Cell-level timings are expressed in days of year and duration in number of days. Cell-level timings and durations in Tree 5 could not be calculated due to incomplete information on the number of mature cells.

In all the studied trees, the number of developing birefringent cells (as noted through TLM) matched the number of lignifying cells counted using CLSM images (Fig. 7), revealing a correspondence between the onset of CML lignification and SCW deposition. Deviations between TLM and CLSM-derived curves were reduced, with the difference in the number of cells oscillating between 0 and 3 tracheids (1.96 ± 1.95 cells on average, Supplementary Table S1). Cell-level timings and durations predicted from individual models (Supplementary Fig. S4) were also remarkably coincident (Fig. 8), with a slight deviation only present for the last-formed latewood cells. A mean offset of only 0.96 d between the 2 curves pointed out the high synchronicity between wall thickening (TLM) and lignification (CLSM) at the cell level, which was conserved along the tree ring and across trees despite the contrasting individual patterns (Fig. 7). On average, tracheids required 25.7 (27.1) d to complete wall thickening (lignification), resulting in a mean difference of 1.9 (Sd = 5.5) d. Maximal durations were observed in latewood (i.e. they took 69 more d to be complete in latewood than in earlywood cells). At the tissue level, wall thickening (TLM) and lignification (CLSM) ranged over the same period, showing coincident onset (day of year - DOY - 138 to 164) and end (DOY 268 to 333) dates (Supplementary Table S1).

## Discussion

### Multimodal imaging as a reliable approach to assess lignification in xylogenesis studies

In this study, we present a comprehensive analysis of tracheid lignification in silver fir by combining spatial information derived from RMS and temporal dynamics reconstructed from TLM and CLSM images. RMS chemical imaging provides molecular insights into cell wall composition by using unique spectral fingerprints to identify compounds being particularly useful for lignin detection (Gierlinger et al. 2012; Guillon et al. 2022; Leng et al. 2022). However, the long time required to obtain and process RMS spectra may limit a widespread application in xylogenesis studies, which typically require observations on numerous samples (Rathgeber et al. 2016). Prior studies have successfully analyzed lignification by using CLSM images (Dickson et al. 2017; Nanayakkara et al. 2019). Yet, the association between native wood fluorescence and lignin content is not always straightforward. Although the distribution pattern of the signal excited by 405 nm light (blue channel) was consistent with the expected for lignin, an optimal lignin autofluorescence excitation would only be achieved by using an ultraviolet (280 to 355 nm wavelength) laser (Dickson et al. 2017; Donaldson 2020). Indeed, while some natural phenolic compounds could fluoresce at similar wavelengths as lignin (Donaldson et al. 2019), artificial compounds (e.g. mounting media) and changes in pH could modify native wood fluorescence (Donaldson 2013; Ding et al. 2020; Donaldson 2020). Furthermore, although fluorescent dyes could enhance lignin visualization (Kitin et al. 2003, 2020; Balzano et al. 2021), it is important to exercise caution when interpreting results due to potential fluorescence quenching caused by suboptimal dye concentrations (Bond et al. 2008). This motivated us to test the correlation between the relative intensity of the RMS band of lignin and the autofluorescence excited by violet/blue light (405 nm) in differentiating xylem. The high correlation yielded between the 2 signals demonstrates that 405-nm light is suitable to excite lignin autofluorescence in silver fir (Fig. 6), validating the use of our CLSM images to monitor lignification. These results underscore the potential of multimodal imagining approaches to reliably analyze xylogenesis.

### Insights into the lignification of silver fir tracheids

Lignin emitted the strongest RMS scattering and fluorescence signal within mature xylem, although its relative concentration varied across cell wall layers. CC and CML emitted stronger fluorescence than the S2 layer (Fig. 4, Supplementary Fig. S2), suggesting a higher lignin content. Limited image resolution challenged distinction between S1 and CML, which could also reflect a relatively similar lignin content in these 2 layers. Lower lignin concentration in S2 than in CML is common in normal conifer wood (Kutscha and Schwarzmann 1975; Donaldson 2001; Schmitt et al. 2003; Agarwal 2006). This may result from highly condensed lignin units formed in the CC and middle lamella due to their rich pectic composition (Terashima and Fukushima 1988; Boerjan et al. 2003; Terashima et al. 2004), but also from associations between lignin and the hemicelluloses that intercalate cellulose microfibrils in the PCW (Terashima et al. 2004). Yet, RMS spectra showed a limited contribution of hemicelluloses in CML, which could be partly related to their noncrystalline structure (Agarwal 1999; Gierlinger et al. 2012). As reported in previous studies (Donaldson 2001; Agarwal 2006), the last formed layer (S3) revealed a more intense lignification than the S2 (Supplementary Fig. S2). Neat S3 boundaries in CLSM images evidenced that S2 was much thicker in latewood than in earlywood, which accounts for the decrease in lignin concentration from earlywood to latewood walls previously reported in conifers (Antonova et al. 2014). Beyond confirming the reliability of our observations, these results suggest that the lignin distribution inferred from our analysis is pervasive across conifer species.

Topochemical information obtained through CLSM and RMS images shed light on cell wall lignification in silver fir. As expected, the spectroscopic signature of lignin prevailed in mature cells, while it was negligible in cambial and enlarging cells (Figs. 2, 3, and 5). Initial lignin-related autofluorescence in differentiating tracheids was observed in CC and CML, spreading later inward into the SCW. This aligns with previous studies showing that lignification initiates at these specific sites (Kutscha and Schwarzmann 1975; Terashima and Fukushima 1988; Donaldson 1991; Fukushima and Terashima 1991; Donaldson 2001; Tobimatsu et al. 2013; Meents et al. 2018). The variable lignin concentration observed across differentiating cells suggests dynamic changes in lignin biosynthesis rates during cell differentiation. The greatest variation in the relative lignin concentration of CML was recorded at initial wall-thickening stages (locations 7 and 8, Fig. 6), which could be attributed to higher lignification rates in middle lamella than in the S2 (Terashima and Fukushima 1988). Lignification rates would then increase again in the S3 (Donaldson 2001; Schmitt et al. 2003; Gričar et al. 2005), as suggested in our trees by the higher lignin content. Heterogeneous lignification across cell wall layers would be accounted by sequential deposition of hemicelluloses and oxidative enzymes (Meents et al. 2018), which are responsible for the polymerization of monolignol deposits secreted by Golgi vesicles (Samuels et al. 2002). Yet, a gradual increase in CML lignin concentration until full cell maturation suggested a certain overlap between CML and SCW lignification, which could be attributed to mobility of monolignols in the polysaccharide matrix (Meents et al. 2018). Indeed, one previous study in balsam fir (*Abies balsamea* (L.) Mill.) reported an overlap between both PCW and SCW lignification, but also between the S3 and the warty layers (Kutscha and Schwarzmann 1975; Schmitt et al. 2003). These results however contrast with those reported in pines, with middle lamella being fully lignified before the start of SCW lignification (Donaldson 1991; Fukushima and Terashima 1991; Donaldson 2001). Thus, whether a variable overlap in lignification between CML and SCW translates into distinct cell-level durations of lignification across species is a question that deserves further investigation.

After cell autolysis, lignification could continue through releases of vacuolar monolignol glucosides into the cell wall (Samuels et al. 2002; Pesquet et al. 2013). Yet, the limited C/L ratio increment recorded between wall-thickening and mature tracheids in CC and CML (Fig. 6) suggests that *postmortem* lignification (if any) would be limited to SCW, particularly S3 and warty layers (Schmitt et al. 2003; Gričar et al. 2005). Moreover, parenchyma and other cell types (e.g. epithelial cells) could become lignified after the completion of the tree ring (Kutscha and Schwarzmann 1975; Donaldson 2001), potentially affecting the degree of lignification of nearby mature tracheary elements (Pesquet et al. 2013; Barros et al. 2015). Although the contribution of neighboring cells to tracheid lignification was not specifically quantified in our samples, tracheids adjacent to parenchyma rays exhibited a comparable degree of lignification to those located at more distant locations within the same radial position (Fig. 4). This suggested that the contribution of neighboring cells to mature tracheid lignification would have a limited quantitative impact.

Cell kinetics reconstructed in our study showed that tracheids spent an average of 30 d in the lignification zone, which is consistent with a previous study reporting several weeks of lignification within a single cell (Savidge and Udagama-Randeniya 1992). However, cell residence times in the lignification zone increased along the tree ring, with much longer periods of lignification in latewood than in earlywood (Fig. 8). Variable lignification periods throughout the tree ring may arise due to an increment in cell wall thickness from early to latewood, but also to seasonal fluctuations in lignin deposition rates. According to Antonova et al. (2007), lignification is more intense during late stages of cell wall formation in earlywood tracheids and during early stages in latewood tracheids, which was attributed to changes in lignin precursors and the carbohydrate matrix throughout the growing season. Latewood cells requiring a long time to become lignified could also result from decreasing temperatures in the fall (Donaldson 2001; Gričar et al. 2005). Recent research has highlighted the significance of temperature in controlling lignification (Crivellaro and Büntgen 2020), which could cease abruptly after exceptionally cold episodes (Schmitt et al. 2003; Piermattei et al. 2020), or being delayed until spring under mild winter conditions (Donaldson 1991; Nanayakkara et al. 2019). However, a full understanding of the climate drivers of lignification would require further research analyzing the intraannual dynamics of lignification under contrasting climate conditions.

### Coordination between SCW deposition and cell wall lignification

Our second hypothesis referred to the time lags between SCW deposition and cell wall lignification. A large temporal offset between the 2 phases was not supported by RMS and CLSM images, as significant amounts of lignin were observed in CC and middle lamella of tracheids at the very initial stages of SCW deposition (Fig. 6). This is in line with previous observations showing that lignin deposition in the CML occurs at an early stage of xylem differentiation (Fukushima and Terashima 1991), being concurrent with the synthesis of a significant fraction of the total cell wall cellulose (Antonova et al. 2007). This idea also finds support from previous studies analyzing lignification through immunoelectron microscopy (Ruel et al. 2002; Joseleau and Ruel 2006), which suggest that lignin impregnates the SCW as soon as the cellulose microfibril matrix is deposited. As previously described in silver fir (Schmitt et al. 2003; Gričar et al. 2005) and pine (Donaldson 1991), wall-thickening tracheids contained a thin unlignified layer adjacent to the lumen (Fig. 4, Supplementary Fig. S2), revealing that cellulose deposition in the SCW starts before its lignification. The limited size of this unlignified layer hints at a short offset between cellulose microfibril deposition and lignin impregnation. However, further exploration would be needed to accurately determine the time lag between the completion of SCW deposition and lignification.

Our quantitative analysis based on cell counts showed a remarkable concurrence between TLM- and CLSM-derived cell kinetics (Figs. 7 and 8), confirming a similar onset for SCW deposition and lignification and refuting our second and third hypotheses. A potential explanation is that lignification likely begins in CC and CML around the time when cellulose microfibrils start to be added to the SCW. In addition, our observations suggested that most lignin may have already been incorporated into the SCW before its completion. Therefore, cellulose and lignin deposition would be highly overlapped at the cell level. These findings align with results from a previous study in Monterey pine (*Pinus radiata* D. Don) showing that lignin autofluorescence and wall thickness across the forming tree ring are highly correlated (Nanayakkara et al. 2019). Similar increases in cell-level durations along the growing season for wall thickening (TLM) and lignification (CLSM) would reflect the strong coregulation between SCW deposition and lignification pointed out in various studies using transgenic plants (e.g. Ruel et al. 2002). While low-lignin transgenic plants were found to have high levels of cellulose matrix disorganization leading to xylem deformation (Ruel et al. 2002; Voelker et al. 2011), advanced lignification in transgenic aspen disrupted PCW extension at early stages of cell enlargement (Grünwald et al. 2002). These lines of evidence suggest that tracheid development requires a fine coordination between the different subprocesses of differentiation. This notion alludes to the fact that different phases of xylogenesis may not only be linked by their temporal sequence but also by their general regulatory mechanisms.

### Implications of coordinated SCW deposition and cell wall lignification for tree-ring science

Our results converge on the idea that SCW deposition and lignification are time coordinated from the cell up to the entire tree-ring level. Moreover, they would imply that most of the cellulose and lignin in a particular tree ring or tree-ring sector are incorporated over comparable time windows, even though a certain amount of lignin could still be deposited after the end of SCW deposition. Despite our study concerning temperate conifers, there are sources of evidence suggesting similar results in temperate angiosperms (Prislan et al. 2009). For instance, overlapped deposition times for cellulose and lignin would explain parallel intraring carbon isotope profiles for cellulose and total wood found in beech (Helle and Schleser 2004), which may however show distinct isotopic signatures due to the different biosynthetic pathways for cellulose and lignin (Gessler et al. 2014). Consequently, it would be possible to deduce intraannual dynamics of lignification by relying on cell-level timings of wall thickening obtained from TLM images. This would ease the obtention of accurate recording time estimates for xylem traits associated with lignin, which may increase our understanding of tree growth responses to environmental cues (Pérez-de-Lis et al. 2022). Moreover, lignification could be more readily accounted for in process-based models of wood formation, which may render more accurate estimations of the seasonal contribution of lignification to woody biomass production.

### Limitations and future directions

Here, timings and durations of lignification are quantified in silver fir tracheids by using a cell-counting approach. However, it is important to highlight that the process of lignification could change across species, cell types, and growing conditions (Schmitt et al. 2003; Gričar et al. 2005). First, lignification could vary across species (Grünwald et al. 2002) likely because of their different monomer compositions. While conifer lignin is primarily composed by guaiacyl (G) units, angiosperms contain a balanced mix of syringyl (S) and G monomers (Boerjan et al. 2003; Vanholme et al. 2010; Guillon et al. 2022), which have different binding affinities with cellulose surfaces (Vermaas et al. 2019). Second, lignification timeframes could vary across cell types (Barros et al. 2015; Balzano et al. 2021). This is the case for angiosperm vessels vs. fibers, which were reported to have different wall deposition and lignification timeframes (Kitin et al. 2003; Prislan et al. 2009), but also for xylem parenchyma vs. tracheids in conifers. In addition, compression wood is characterized by a more heavily lignified S2 layer (Donaldson 2001), likely because of a longer duration of lignification in that specific layer (Fukushima and Terashima 1991). Finally, lignification can be affected by multiple other factors that have not been addressed in our study, such as wounding and pathogen infection or climate conditions (Donaldson 2002; Crivellaro and Büntgen 2020; Ranade et al. 2022), which could impact key wood traits (i.e. resistance to cavitation) via altered lignification timeframes (Pereira et al. 2018). Therefore, we advocate for extending the proposed approach to different species and growing conditions, which could also pave the way for a more accurate consideration of xylogenesis phases in wood formation models.

## Conclusion

Despite the renewed attention xylogenesis has received in recent decades, the temporal coordination between cell SCW deposition and cell wall lignification is still largely ignored, which hinders our understanding of the links between cell wall formation and related xylem traits. Here, we utilized multimodal imaging for a xylogenesis investigation, aiming to compare the kinetics of wall thickening (i.e. SCW deposition and cell wall lignification) and lignification. RMS chemical images and CLSM images showed that lignification of the middle lamella starts along with the secondary wall deposition, which may finish once most of the lignin present in the cell wall has already been incorporated. This was in part confirmed by the comparable timings and durations obtained for wall thickening (TLM) and lignification (CLSM). Taken together, these results suggest that most cellulose and lignin are deposited in the xylem over similar timeframes. Our findings endorse utilization of TLM-derived wall-thickening timings to infer cell wall lignification dates while suggesting that the environmentally driven repression of lignification resulting in blue rings may be already triggered in incipient wall-thickening stages. Moreover, the large overlap between cellulose and lignin incorporation into the tree-ring structure could assist in understanding how isotope fractionation associated with lignin biosynthesis affects the environmental and physiological signals captured in whole-wood isotope data sets. Finally, although our study offers perspectives in xylogenesis research, a comprehensive understanding of lignification would demand further insights into the cell wall lignification rates and potential variations across species, cell types, and growing conditions. This objective can only be achieved by combining different imaging and analytical tools aimed at investigating the underlying processes and their implications for tree modeling and function.

## Materials and methods

### Experimental design and sample processing

The samples used in this study were collected in a mixed stand located in Walscheid (48°38′N, 7°09′E, 370 m a.s.l.), in the Vosges mountains (northeast France). Temperate climate conditions prevail at the study area, but there is a strong continental influence. Microcores containing the last forming ring were collected every week (35 sampling dates) from the main stem of 5 silver fir (*A. alba* Mill.) trees in 2010 by using a Trephor tool.

Microcores were dehydrated, embedded in paraffin, and sectioned with an automated rotary microtome (Leica RM2255, Wetzlar, Germany) to obtain 6- to 8-µm-thick transverse wood microsections, which were stained with Cresyl violet and mounted on glass slides (see Cuny et al. 2012 for further detail). CSLM and RMS analyses were carried out on additional microsections obtained from the remaining wood samples in the paraffin blocks. Two successive immersions in D-limonene and absolute alcohol were carried out to remove the remaining paraffin attached to the microsections, which were permanently fixed with Histolaque (CellPath Ltd, Newtown, United Kingdom) on glass slides pretreated with glycerol albumin. No Cresyl violet or any other dye was applied on these samples.

### TLM observations

Cresyl violet–stained microsections were observed under a TLM to count the number of tracheids in 4 main differentiation zones: cambial, cell enlargement, wall thickening and lignification, and mature. Cambial and enlarging cells had thin violet walls, but the latter had a wider diameter. Wall-thickening cells had the same dimensions as those in the enlarging zone, but their walls were birefringent under polarized light (Fig. 4). Mature tracheids were identified thanks to their empty lumens and fully blue-colored walls. Cells in each zone were counted along 3 radial files per sample and averaged (see Cuny et al. 2012 for further details). Resin ducts and epithelial cells were disregarded when present within a cell radial file (Fig. 4).

### RMS spectra and hyperspectral image acquisition

We used RMS to analyze the cell wall composition within a 19-µm-thick unstained microsection collected on DOY 215. RMS spectra and chemical maps were obtained at CC and CML over 3 main microsection zones: mature xylem zone (1 location), cambial zone (1 location), and differentiation zones (9 locations that comprised both enlarging and wall-thickening cells). CML is composed of middle lamella and PCW, with potential contributions from S1 of the SCW.

RMS spectra and hyperspectral maps were collected within a range of 652 to 3,119 cm^−1^ with a Renishaw inVia Qontor spectrometer equipped with a Leica confocal microscope. We recorded measurements using either an Olympus 80× objective or a Leica 50× long-distance objective. The instrument was equipped with an edge filter to eliminate the Rayleigh scattering, 1,200 lines per millimeter grating, and the CCD Renishaw Centrus detector. The excitation source was at 532 nm, and the laser power was ca. 5 mW at the sample. The acquisition was performed with 4 accumulations of 10 (50× objective) or 20 (80× objective) seconds using the Live Track option, and the step was set to 1 *µ*m. Cosmic rays were removed, and spectra were baseline corrected with a polynomial function from the Wire 5.5 software. The noise filter from Wire 5.5 software was applied on the spectra from the maps using 4 components. For RMS hyperspectral maps, we used the Gwyddion software (Nečas and Klapetek 2012) to obtain stacked image files and to extract the calibration of Raman shift numbers corresponding to stack slice numbers.

The scattering RMS bands were assigned in accordance with Leng et al. (2022). RMS chemical images were obtained by measuring the integrated intensities from signal to baseline in the ranges 995 to 1,009 (centered at 1,003), 1,085 to 1,105 (centered at 1,096), 1,112 to 1,173 (centered at ∼1,127), and 1,592 to 1,604 (centered at 1,599) cm^−1^ for Histolaque proteins, cellulose, cellulose and hemicelluloses, and lignin, respectively. Coniferyl alcohol and coniferyl aldehyde were assumed not to substantially contribute to the integrated signal for lignin (Bock and Gierlinger 2019). Although the intensity of the cellulose band centered at 1,096 cm^−1^ could vary based on the orientation of the cellulose backbone, potentially affecting cellulose visualization, it directly corresponds to the pure C–O–C stretching of cellulose. Furthermore, since we only examined 1 microsection, any impact from these fluctuations would be reduced. On the other hand, RMS scattering has a weak effect compared to fluorescence that may occur during spectra recording. A strong fluorescence background might then become the dominant element, precluding the detection of the RMS scattering signal (Agarwal 1999). Indeed, we were not able to analyze some areas of the wood sample because of the saturation of the RMS detector by background fluorescence (Supplementary Fig. S5).

### CLSM image acquisition

We observed 6- to 8-µm-thick unstained microsections under a CLSM equipped with 405 (violet/blue) and 488 nm (blue) excitation laser beams and GaAsP PMT detector allowing for spectral imaging, coupled to ZEN 2.1 lite black software (Zeiss LSM 780 inverted Axio Observer, Oberkochen, Germany). We analyzed a total of 53 samples from 11 regularly spaced sampling dates. Autofluorescence emission spectra of cell wall components in the 410 to 695 nm bandwidth were acquired independently in both mature and differentiation zones, as well as in the cambial zone and phloem using lambda acquisition mode with GaAsP spectral array detector with either 405- or 488-nm laser beams. Emission spectra were recorded and further utilized to generate spectral deconvolution images of a cell transect from phloem to mature xylem. This process employed the online fingerprinting mode with dual excitation of 405 and 488 nm beams simultaneously. Images of the last forming ring were acquired with 20× 0.8 NA and 50× 0.95 NA objectives, while a pseudocolor image processing was applied to the different detector channels. We employed a laser power setting of 1% and 1.8% for 405- and 488-nm beams and a pinhole diameter of 1.01 airy units (equivalent to 1.8 *µ*m section), while gain, offset, and other imaging parameters were set in each sample in the fully mature zone of the previous ring. The final images were obtained after stitching together a series of images using a combination of tile scan (15% overlap) and Z stack functions taken at 2.06-*µ*m-deep intervals along the whole sample thickness. Then the maximum intensity projection method was applied to create final composite images (Fig. 4). Spectral CLSM images obtained from the different detector channels were combined into a single composite image in ZEN 2.1 lite black software.

### Analysis of the lignin content in the cell wall

Since the relative concentration of different structural cell wall compounds can be inferred through the scattering intensity of their respective bands (Gierlinger et al. 2012), we quantified the RMS intensity for the cellulose (1,085 to 1,105 cm^−1^) and lignin (1,592 to 1,604 cm^−1^) bands across the selected locations in mature and differentiating xylem. We then compared the relative RMS intensity with the relative intensity of the autofluorescence signal excited at 405 nm quantified on the same locations in the corresponding CLSM image. This was possible because spatial variation of lignin-related fluorescence intensity correlates to its relative abundance (Guillon et al. 2022). The autofluorescence signal intensity was measured at the 7th channel (464 to 473 nm detection range), where the emission signal peaked within the lambda mode spectra (Supplementary Fig. S6). The resolution of CLSM images (0.18 *µ*m × pixel^−1^) was harmonized with that of the RMS hyperspectral maps (1 *µ*m × pixel^−1^) to ensure consistent image comparisons. We then obtained the intensity of both fluorescence and Raman signals on the selected locations (2 × 2 *µ*m each) by averaging the signal intensity of 4 pixels. The orientation of cellulose microfibrils in locations with potential occurrence of the S1 was assumed to be comparable across cells.

### Quantitative data analysis

To test whether the autofluorescence signal excited at 405 nm reflected the presence of lignin in the cell wall, we compared its relative intensity with that of the RMS scattering for lignin at the same locations. The alignment of RMS hyperspectral maps and autofluorescence images was done semiautomatically using bUnwrapJ registration plugin (Arganda-Carreras et al. 2006) in FIJI/ImageJ (Schneider et al. 2012). The agreement between CLSM and RMS relative signal intensities was verified through linear regression. Moreover, changes in lignin concentration in CC and CML between mature and differentiating xylem were evaluated through the C/L ratio, which was calculated from the corresponding integrated Raman intensities for the cellulose and lignin bands in each of the selected locations.

After verifying the correspondence between wood autofluorescence signal and lignin content, we used CLSM images to compute the number of lignifying cells, which were defined as those showing the 2 fluorescent emission signals in their walls (excited by the 405 and 488 nm laser beams; Fig. 4). First, composite images were assembled by using linear unmixing to clear up the signal from several fluorophores with overlapped emission spectra. In this manner, each pixel displayed only the strongest fluorescent emission for that pixel (Fig. 4). Composite images were subsequently split into separate RGB channels, and cells were counted across 3 radial files and averaged for each microsection. The region of interest for cell counting was delimited by (i) the youngest xylem cell in which fluorescent emission excited at 405 nm was prevalent in any of the wall layers (blue box in Fig. 4) and (ii) the oldest cell in which fluorescent emission excited at 488 nm was prevalent in any of the wall layers (yellow box in Fig. 4).

We described the intraannual dynamics of cell wall thickening (i.e. SCW deposition and cell wall lignification) and lignification for each tree by alternatively using xylem cell counts obtained from TLM and CLSM images. Generalized additive models (GAMs) were adjusted to both data sets by using a cubic regression spline, while a shape-constrained additive model (SCAM) with a quasi-Poisson distribution was fitted to mature cell counts obtained from TLM images. Model-predicted curves were used to infer cell-level timings and durations of cell wall thickening and lignification, obtaining estimations for their onset and end at the tree-ring level. GAM and SCAM calculations were performed in R version 3.5.0 (R Core Team 2018) by using the packages “mgcv” (Wood 2011) and “scam” (Pya and Wood 2014), respectively. In addition, the FIJI/ImageJ software was used to convert multispectral files into single-image files with the selected fluorescence emission, to determine the radial cell count in TLM and CLSM images, and to quantify both Raman and fluorescence signal intensities.

## Supplementary Material

kiae203_Supplementary_Data

## Data Availability

The data underlying this article will be shared on reasonable request to the corresponding author.

## Supplementary data

The following materials are available in the online version of this article.

**Supplementary Figure S1.** RMS exploration of mature xylem.

**Supplementary Figure S2.** Detailed autofluorescence images of transverse wood microsections.

**Supplementary Figure S3.** RMS exploration of differentiating xylem.

**Supplementary Figure S4.** GAM curves for the number of wall-thickening and lignifying tracheids.

**Supplementary Figure S5.** Luminescence and fluorescence saturation.

**Supplementary Figure S6.** Autofluorescence emission spectra in successive lambda planes after excitation by the 405-nm laser beam.

**Supplementary Table S1.** Deviation between wall thickening (TLM) and lignifying (CLSM) cell counts and predicted dates for the onset and cessation of wall thickening (TLM) and lignification (CLSM) according to GAMs.
