# Supplementary material for: Multimodal imaging analysis in silver fir reveals coordination in cellulose and lignin deposition
Source: Plant Physiol. 2024 Apr 9;195(3):2428–42. doi: 10.1093/plphys/kiae203 (PMC11213250; doi:10.1093/plphys/kiae203)
Supplement: kiae203_Supplementary_Data [file kiae203_supplementary_data.zip › PP2023RA01883R1_Supplemental_Material.pdf]

Supplementary Material:

Multimodal imaging analysis in silver fir reveals coordination in cellulose and lignin deposition

Gonzalo Pérez-de-Lis, Béatrice Richard, Fabienne Quilès, Aurélie Deveau, Ignatius-Kristia Adikurnia, Cyrille B. K. Rathgeber

Supplementary Figure S1. Raman microspectroscopy exploration of mature xylem. **(A)** Transmitted light microscopy image and **(B–D)** Raman microspectroscopy chemical images based on the specific bands for **(B)** cellulose and hemicelluloses (integrated intensities between 1112 and 1147 cm^{-1}), **(C)** cellulose (integrated intensities between 1085 and 1105 cm^{-1}) and **(D)** Histolaque (integrated intensities between 995 and 1009 cm^{-1}) on the same region of interest. The chemical images were obtained in the same zone as in **Figure 2**.

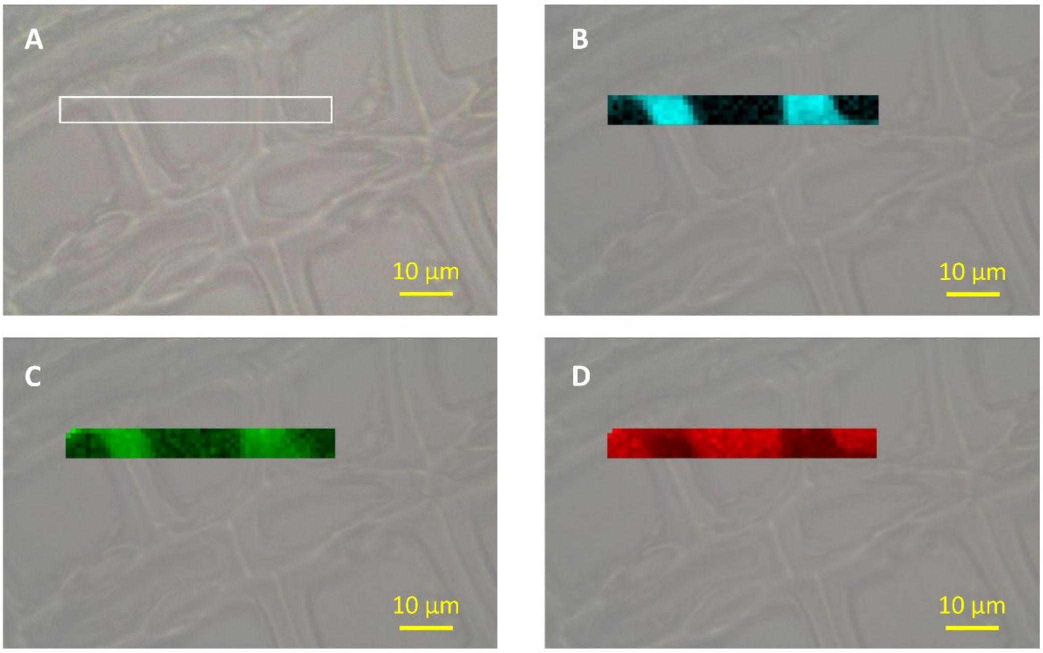

Supplementary Figure S2. Detailed autofluorescence images of transverse wood microsections. **(A)** composite image showing the emission signal of two different fluorophores within the cell walls of differentiating tracheids, and **(B)** gray-scale image of the signal excited at 405 nm revealing distinct signal intensity between S2 and both S3 and compound middle lamella (middle lamella and primary cell wall) in mature tracheids. Images **A** and **B** were obtained on the same sample as in **Figure 4**.

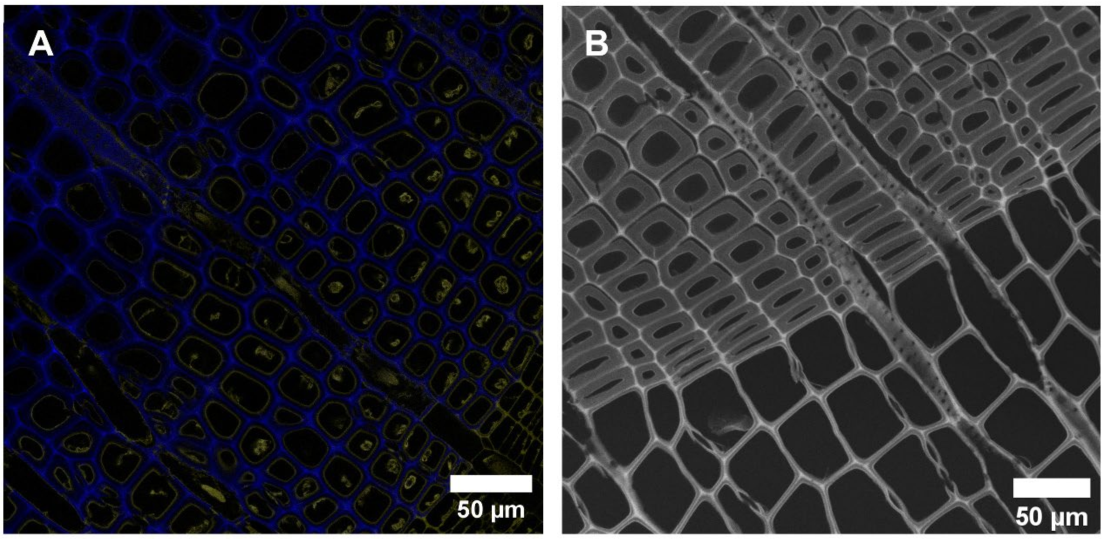

Supplementary Figure S3. Raman microspectroscopy exploration of differentiating xylem. **(A)** Transmitted light microscopy image with locations and regions of interest where Raman spectra and hyperspectral maps were respectively obtained. **(B–D)** Raman chemical images based on the specific bands for **(B)** cellulose and hemicelluloses (integrated intensities between 1112 and 1147 cm^{-1}), **(C)** cellulose (integrated intensities between 1085 and 1105 cm^{-1}) and **(D)** Histolaque (integrated intensities between 995 and 1009 cm^{-1}) on the same regions of interest. The chemical images were obtained in the same zone as in **Figure 5**.

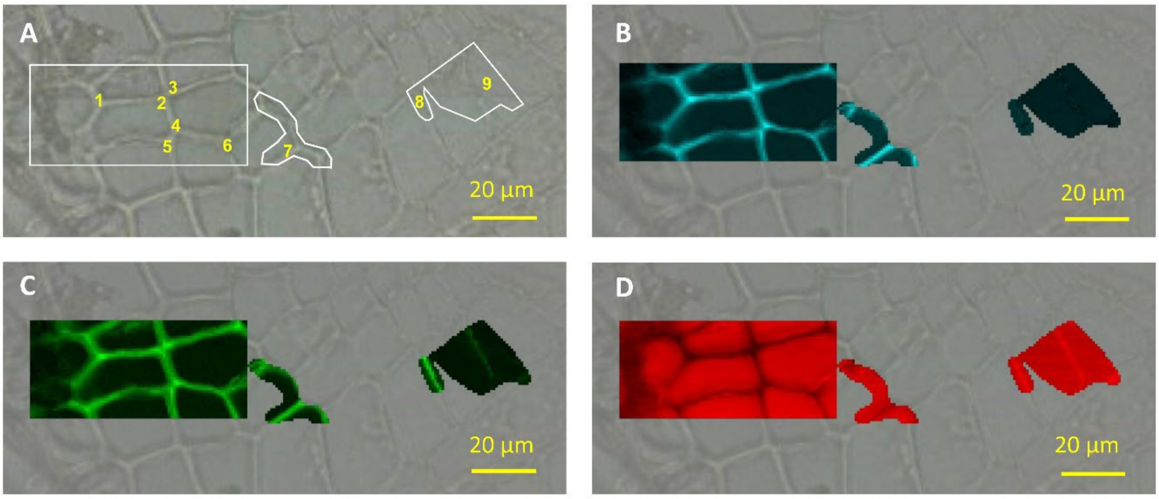

Supplementary Figure S4. Generalized Additive Model curves for the number of wall-thickening and lignifying tracheids. Wall-thickening (red circles) and lignifying (purple triangles) cell counts were obtained in five trees through transmitted light and confocal laser scanning microscopy imaging, respectively. Predicted curves are shown for each phase and tree. Dots show cell counts used to fit the models, which are the average of three radial files for each date and phase.

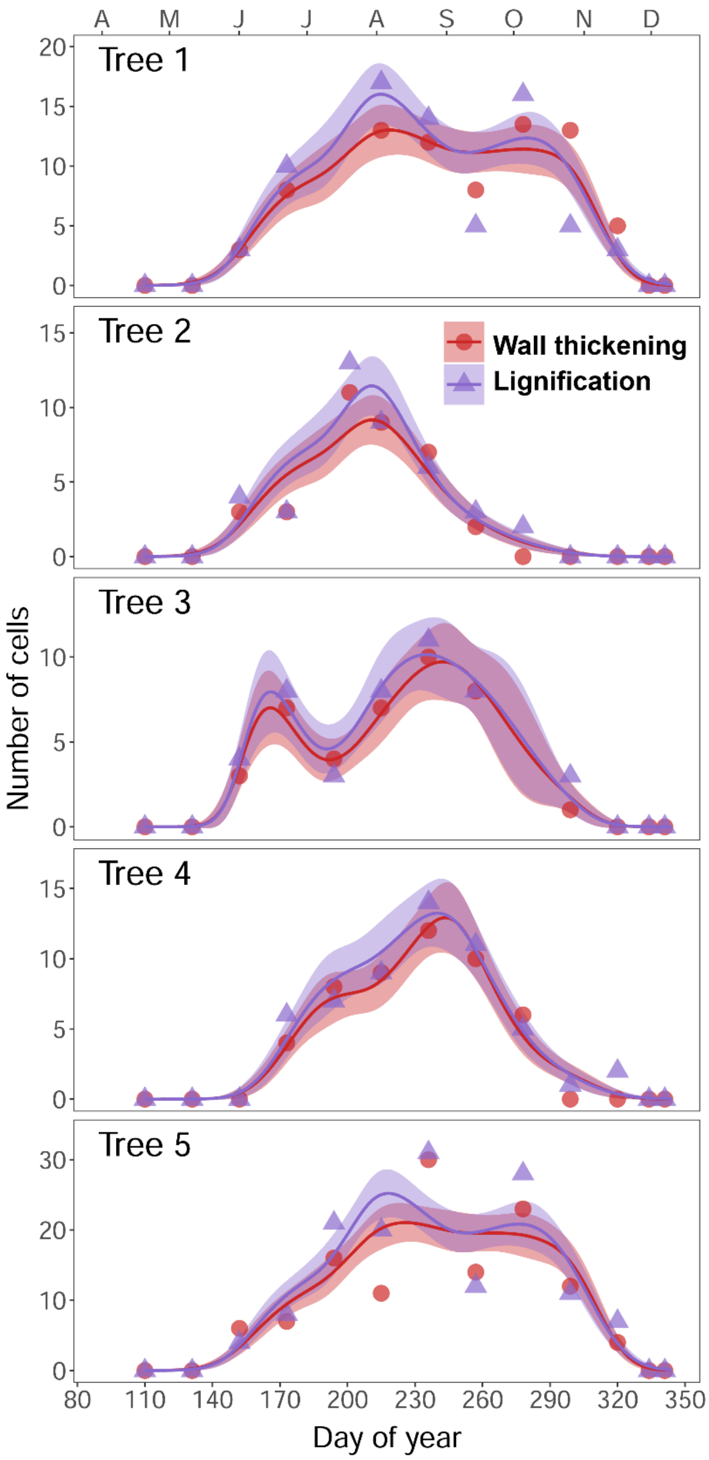

Supplementary Figure S5. Luminescence and fluorescence saturation. Transmitted light microscopy image of differentiating xylem and the same zone showing luminescence **(A)** and fluorescence **(B)** saturation (green areas). Each image in panels **A** and **B** is composed of four different images.

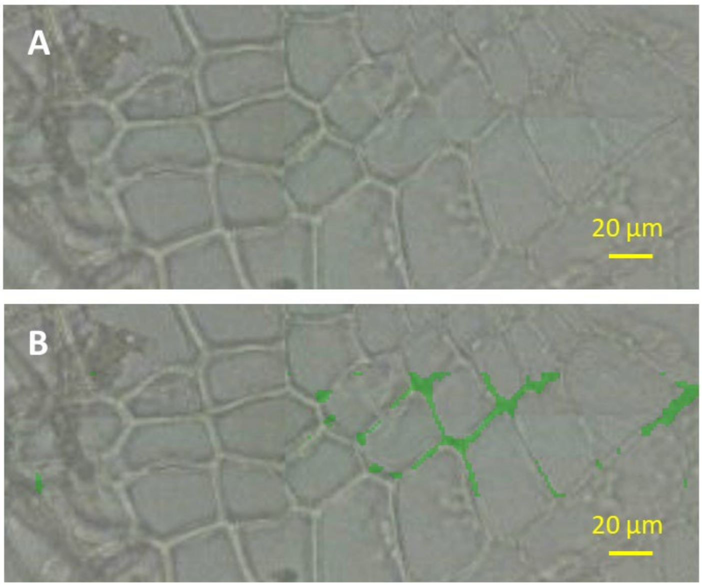

Supplementary Figure S6. Autofluorescence emission spectra in successive lambda planes after excitation by the 405 nm laser beam. Emission is provided in arbitrary units (AU) for enlarging (blue dots) and wall-thickening (magenta triangles) tracheids within the differentiation zone. Each point is associated to one emission channel consisting of nine successive wavelengths.

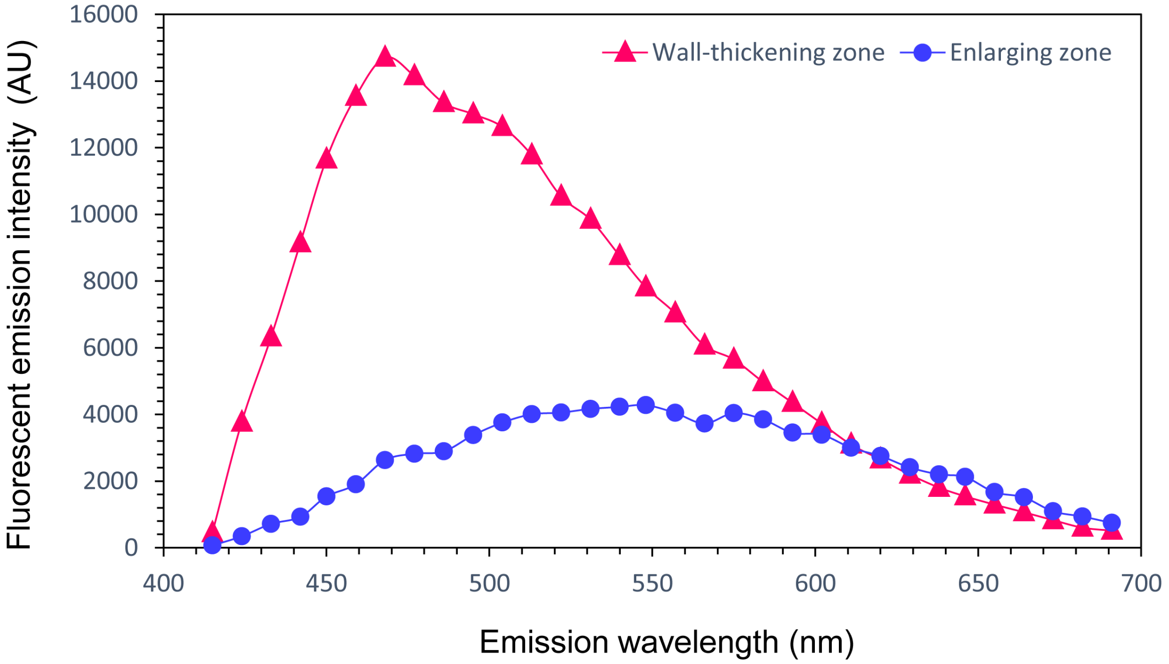

Supplementary Table S1. Deviation between wall thickening (transmitted light microscopy, TLM) and lignifying (confocal laser scanning microscopy, CLSM) cell counts and predicted dates for the onset and cessation of wall thickening (TLM) and lignification (CLSM) according to Generalized Additive Models.

Tree	**Mean deviation in no. of tracheids between TLM and CLSM ± SD**	**Date of onset (DOY)**		**Date of cessation (DOY)**	
		TLM	CLSM	TLM	CLSM
1	0.69 ± 0.87	138 – 146	138 – 147	323 – 329	324 – 331
2	0.40 ± 0.64	140 – 150	141 – 150	268 – 286	269 – 287
3	0.37 ± 0.47	141 – 149	141 – 148	296 – 311	296 – 310
4	0.42 ± 0.62	154 – 164	154 – 163	299 – 315	299 – 315
5	1.03 ± 1.33	137 – 146	138 – 146	325 – 331	326 – 333
