# Supplementary material for: Multimodal imaging analysis in silver fir reveals coordination in cellulose and lignin deposition
Source: Plant Physiol. 2024 Apr 9;195(3):2428–42. doi: 10.1093/plphys/kiae203 (PMC11213250; doi:10.1093/plphys/kiae203)
Supplement: kiae203_Supplementary_Data [file pp.01883.2023-s03.pdf › pp.01883.2023-s03.pdf]

Plant Physiology®

Are cellulose and lignin deposition coordinated? A multimodal imaging analysis of xylogenesis in silver fir

Gonzalo Pérez-de-Lis, Béatrice Richard, Fabienne Quilès, Aurélie Deveau, Ignatius-Kristia Adikurnia, and Cyrille Rathgeber

Review Timeline:

Submission Date:	29-Nov-2023
Editorial Decision:	01-Feb-2024
Revision Received:	18-Mar-2024
Accepted:	18-Mar-2024

01-Feb-2024

Dr. Gonzalo Pérez-de-Lis
Universidade de Santiago de Compostela
Lugo
Spain

RE: Are wall thickening and lignification coordinated? A multimodal imaging analysis of xylogenesis in silver fir

Dear Dr. Pérez-de-Lis:

We have received reviews of your manuscript, which you may view at the end of this letter. On the basis of the advice received and my own evaluation, I believe that, pending revision, this article may ultimately be acceptable for publication in Plant Physiology.

Both reviewers have raised some comments for you to address. Additionally, they recommend that the MS be proofed by someone whose first language is English- and I concur!

When you are ready to submit your revision, click the below link and find the red arrow under your Author Tasks heading for this revision submission.

If you want to visit this version of the manuscript, you may click the link below and then click on the folder that reads "Post Decision Manuscripts".

Link Not Available

When uploading the revised version of this article, please be sure to include as complete an answer as possible to the referees' criticisms during the first round of reviews in the "response to reviewers" section.

Plant Physiology considers a revision for up to 60 days after a manuscript has been provisionally accepted; limited extension of this timeframe is at the discretion of the Monitoring Editor. A reminder notice will be sent to you 2 weeks before the deadline, at which time you may choose to contact the editorial office to request an extension; if so, please provide a justification for your request. After the expiration of this deadline, the article would need to be submitted as a resubmission and would be handled as such.

Thank you very much for giving us an opportunity to review this work. I look forward to receiving the next version.

Sincerely,
Board of Editors, Plant Physiology

Want to add this revision deadline to your calendar? Click below!

--- Reviewer comments:
Reviewer #1 (Comments for the Author):

This manuscript considers the early stages of lignification and cell wall thickening in wood formation using confocal fluorescence and raman spectroscopy. The authors conclude that both processes are linked in fir trees although there is scope to investigate variability among species site and tree age in future studies. More interestingly the authors confirm that the very early fluorescence associated with tracheids just after the completion of cell expansion is likely to be lignin as confirmed by Raman spectroscopy. This had been observed in earlier studies but was speculative. This confirms that fluorescence is a very sensitive method for detecting the very early stages of lignification and that perhaps other methods that report the start of lignification later in development are less sensitive. The manuscript needs to be carefully checked for spelling and grammatical errors. At line 96

an important reference was missed from the citations - 32. Tobimatsu Y, Wagner A, Donaldson L, Mitra P, Niculaes C, Dima O, Kim J-I, Anderson N, Loque D, Boerjan W, Chapple C, Ralph J. 2013: Visualization of plant cell wall lignification using fluorescence-tagged monolignols. Plant Journal 76: 357-366.
At line 205 cellulose in the primary wall is randomly oriented, there is no cellulose in the middle lamella so this needs clarification. At line 305 extractive tend to fluoresce at longer wavelengths or not at all - you could cite Donaldson LA, Singh A, Raymond L, Hill S, Schmitt U. 2019: Extractive distribution in Pseudotsuga menziesii: effects on cell wall porosity in sapwood and heartwood. IAWA J. 40: 721-740.
Does coniferaldehyde or small oligomers fluoresce or can Raman spectroscopy differentiate this from lignin?

Reviewer #3 (Comments for the Author):

Dear authors,
Thank you for your interesting work aimed to elucidate the synchronicity of wall thickening and lignification processes during the differentiation of the tracheids in the wood of Abies alba on intra-annual level by combining te TLM, RMS and CLSM.

I have some general comments to include them in the revised manuscript, especially introduction, and discussion.
Some specific comments and recommendations are also included in the attached commented *pdf of the manuscript. I have signed my comments as revA.

I partly agree with you that "...the dynamics of tracheid lignification is generally overlooked in xylogenesis studies, which hampers our understanding of environmental drivers..."(Lines 15-17)

To my opinion, this is because the standardized xylogensis studies consider "the cell wall deposition and lignification" as one phase. However, the wood anatomy slides with cross-sections of the wood used for xylogensis studes are usually stained so that the two phases (cell wall deposition and lignification) can be distinguished, so they could be registered separately. I agree with you that your study gives basic knowledge needed to better interpret the two phases.

The presumable question if "wall thickening and lignification are synchronous" was to my opinion already explained in detail in Abies alba (and some other softwoods and hardwoods) by using transmission electron microscopy (TEM) with adequate fixation and contrasting with the potassium permanganate according to Donaldson (1992, https://brill.com/view/journals/iawa/13/4/article-p381_3.xml) and especially the combination of TEM and UV-micsrospectrophotometry (UMSP) (e.g., Koch & Kleist, 2001, https://www.degruyter.com/document/doi/10.1515/HF.2001.091/html, Schmitt et al., 2003 https://brill.com/view/journals/iawa/24/1/article-p41_4.xml, Gričar et al. 2005 https://doi.org/10.1093/aob/mci112). I suggest you to include this in your manuscript.

The previous results on Abies alba (and some other species) showed the following:
TEM with excellent resolution and possibility of large magnification on samples taken at various locations in the forming tree ring or at different sampling times or from trees with different (phenological) condition can clearly show the parts of the cell wall undergoing thickening (not yet lignified) and the parts with lignification. TEM gives qualitative aspect based on darker grey colour. When TEM observations are supported by UMSP we obtain the degree of lignification evaluated by UV absorbance (quantitative aspect).
This techniques previously used on Abies alba have clearly shown the earliest lignification in CC (cell corners) and CML (compound) middle lamella progressing to layers S1, S2, S3 and (in Abies alba) warty layer (Schmitt et al., 2003, Gricar et al., 2005). Lignifiation always followed the cell wall thickening with some delay from the CML towards the lumen. The methodology enabled also observations on hardwoods like Fagus sylvatica, observation of different types of lignin, and duration of cell wall deposition and lignification (Prislan et al. 2009, https://doi.org/10.1515/HF.2009.079).
As you mention, the cell wall formation is completed and cell maturation is achieved when the cell dies (programmed death) and the plasma disintegrates (no more plasma in the lumen). After the cell death and autolysis, lignin precursors (monolignols) cannot be synthetized any more, however their polymerization to form a lignin macromolecule within the cell wall may still continue (as you describe in lines 384-385 ... After cell autolysis, lignification could continue through releases of vacuolar mognolinol glucosides into the cell wall (Samuels et al., 2002; Pesquet et al., 2013).

"Abies alba L." is not correct- please change the name to "Abies alba Mill." (everywhere)

Comments to Figure 2.
Fig. 2 a, b please mark where the cambium is
Abies alba on Fig. 2c contains traumatic resin canals (TRC). TRC occurrence is due to mechanical injury. This is an exceptional event, therefore TRC are not a part of normal tree ring formation. Lignification of their epithelial cells has another dynamics than normal xylogenesis.

Lines 300-315, please check the wording- it should be precise and careful
there is synchronity among wall thickening and lignification- but the lignification is more or less delayed and ends later than the

thickening.

RESPONSE TO REVIEWERS:

Below, we provide our point-by-point responses to the reviewers' comments (highlighted in bold). Comments from reviewer#3 in the annotated pdf file are included as "minor comments".

Reviewer #1 (Comments for the Author):

This manuscript considers the early stages of lignification and cell wall thickening in wood formation using confocal fluorescence and raman spectroscopy. The authors conclude that both processes are linked in fir trees although there is scope to investigate variability among species site and tree age in future studies. More interestingly the authors confirm that the very early fluorescence associated with tracheids just after the completion of cell expansion is likely to be lignin as confirmed by Raman spectroscopy. This had been observed in earlier studies but was speculative. This confirms that fluorescence is a very sensitive method for detecting the very early stages of lignification and that perhaps other methods that report the start of lignification later in development are less sensitive.
We thank the reviewer for the positive feedback.

The manuscript needs to be carefully checked for spelling and grammatical errors.
The manuscript has been revised by a professional English proofreader.

At line 96 an important reference was missed from the citations - 32. Tobimatsu Y, Wagner A, Donaldson L, Mitra P, Niculaes C, Dima O, Kim J-I, Anderson N, Loque D, Boerjan W, Chapple C, Ralph J. 2013: Visualization of plant cell wall lignification using fluorescence-tagged monolignols. Plant Journal 76: 357-366.
This suggestion is very much appreciated. The reference has been included in both the Introduction and Discussion given its relevance.

At line 205 cellulose in the primary wall is randomly oriented, there is no cellulose in the middle lamella so this needs clarification.
We thank the reviewer for pointing out this error. Raman spectra were obtained at cell corner and compound middle lamella (not middle lamella), but we could not completely discard potential contributions from S1 (with oriented cellulose microfibrils) to the profiles obtained in wall thickening cells. Although the potential contribution of S1 was mentioned in lines 146-147, we replaced "middle lamella" with "locations with potential -occurrence of the S1" for further clarification in lines 208-209.

At line 305 extractive tend to fluoresce at longer wavelengths or not at all - you could cite Donaldson LA, Singh A, Raymond L, Hill S, Schmitt U. 2019: Extractive distribution in Pseudotsuga menziesii: effects on cell wall porosity in sapwood and heartwood. IAWA J. 40: 721-740.
We thank the reviewer for this suggestion. According to that previous study (Fig 4C), it appears that some phenolic compounds may have fluorescence emissions at similar wavelengths as lignin in our study (513-575 nm). Accordingly, we replaced "extractives" with "compounds".

Does coniferaldehyde or small oligomers fluoresce or can Raman spectroscopy differentiate this from lignin?
Raman spectra can differentiate between lignin precursors and lignin despite the very low amount of the formers (<5%). Both lignin precursor monolignols coniferyl alcohol and coniferyl aldehyde have characteristic C=C stretching bands at 1653 cm^{-1} and 1623 cm^{-1} respectively (Bock and Gierlinger 2019,

Bock et al., 2020). These bands are outside the integrated region for lignin (1592cm^{-1} to 1604 cm^{-1}). This is now mentioned in lines 164-165 of the revised version. On the other hand, we do not think that coniferyl alcohol or coniferyl aldehyde could be confused with lignin using fluorescence under the 405-nm excitation. Although they contain by a benzenic ring that is susceptible to fluoresce, previous studies concerning lignin precursors have used fluorescent probes to visualize monolignols (Tobimatsu et al., 2011; 2013), which suggests that their natural autofluorescence intensity is likely too weak to be detected.

Bock P, Gierlinger N (2019) Infrared and Raman spectra of lignin substructures: Coniferyl alcohol, abietin, and coniferyl aldehyde. J Raman Spectrosc 50: 778–792.
Bock P, Nousiainen P, Elder T, Blaukopf M, Amer H, Zirbs R, Potthast A, Gierlinger N (2020) Infrared and Raman spectra of lignin substructures: Dibenzodioxocin. J Raman Spectrosc 51: 422–431
Tobimatsu Y, Davidson CL, Grabber JH, Ralph J (2011) Fluorescence-tagged monolignols: Synthesis, and application to studying in vitro lignification. Biomacromolecules 12: 1752–1761
Tobimatsu Y, Wagner A, Donaldson LA, Mitra P, Niculaes C, Dima O, Kim JI, Anderson N, Loque D, Boerjan W, et al (2013) Visualization of plant cell wall lignification using fluorescence-tagged monolignols. Plant J 76: 357–366

Reviewer #3 (Comments for the Author):

Dear authors,

Thank you for your interesting work aimed to elucidate the synchronicity of wall thickening and lignification processes during the differentiation of the tracheids in the wood of Abies alba on intra-annual level by combining te TLM, RMS and CLSM. I have some general comments to include them in the revised manuscript, especially introduction, and discussion. Some specific comments and recommendations are also included in the attached commented *pdf of the manuscript. I have signed my comments as revA.
We appreciate the positive feedback.

I partly agree with you that "...the dynamics of tracheid lignification is generally overlooked in xylogenesis studies, which hampers our understanding of environmental drivers..."(Lines 15-17). To my opinion, this is because the standardized xylogensis studies consider "the cell wall deposition and lignification" as one phase. However, the wood anatomy slides with cross-sections of the wood used for xylogensis studes are usually stained so that the two phases (cell wall deposition and lignification) can be distinguished, so they could be registered separately. I agree with you that your study gives basic knowledge needed to better interpret the two phases.
We concur with the reviewer's observation. We thoroughly revised and improved this part to clarify the reasons why there is a lack of distinction between secondary cell wall deposition and cell wall lignification in xylogenesis studies (lines 78-86 of the revised manuscript).

The presumable question if "wall thickening and lignification are synchronous" was to my opinion already explained in detail in Abies alba (and some other softwoods and hardwoods) by using transmission electron microscopy (TEM) with adequate fixation and contrasting with the potassium permanganate according to Donaldson (1992, https://brill.com/view/journals/iawa/13/4/article-p381_3.xml) and especially the combination of TEM and UV-micsrospectrophotometry (UMSP) (e.g., Koch & Kleist, 2001, https://www.degruyter.com/document/doi/10.1515/HF.2001.091/html, Schmitt et al., 2003 https://brill.com/view/journals/iawa/24/1/article-p41_4.xml, Gričar et al. 2005 https://doi.org/10.1093/aob/mci112). I suggest you to include this in your manuscript. The previous

results on Abies alba (and some other species) showed the following: TEM with excellent resolution and possibility of large magnification on samples taken at various locations in the forming tree ring or at different sampling times or from trees with different (phenological) condition can clearly show the parts of the cell wall undergoing thickening (not yet lignified) and the parts with lignification. TEM gives qualitative aspect based on darker grey colour. When TEM observations are supported by UMSP we obtain the degree of lignification evaluated by UV absorbance (quantitative aspect).

We thank the reviewer for the information provided on TEM (part of which is now included in the Introduction and the Discussion). On the other hand, although we agree with the reviewer that there are previous works analyzing lignification in conifers, we reconstructed for the first time their kinetics and compared them to those of wall thickening obtained through TLM. We further clarify this in the Discussion (please, see our reply to minor comment in line 464). On the other hand, to have enough room for the new references, we removed two redundant citations (Rossi et al., 2006 and Crivellaro et al., 2022).

This techniques previously used on Abies alba have clearly shown the earliest lignification in CC (cell corners) and CML (compound) middle lamella progressing to layers S1, S2, S3 and (in Abies alba) warty layer (Schmitt et al., 2003, Gricar et al., 2005). Lignifiation always followed the cell wall thickening with some delay from the CML towards the lumen. The methodology enabled also observations on hardwoods like Fagus sylvatica, observation of different types of lignin, and duration of cell wall deposition and lignification (Prislan et al. 2009, https://doi.org/10.1515/HF.2009.079).

We reorganized ideas in the second paragraph of the Introduction to better explain the process of xylem formation, including a mention to the warty layer. We also emphasized the idea that lignification follows cellulose deposition within the secondary wall, but also that there is not an agreement regarding the time lag between lignification and SCW deposition at the cell level. Moreover, Prislan et al. (2009) is now cited in the Discussion.

As you mention, the cell wall formation is completed and cell maturation is achieved when the cell dies (programmed death) and the plasma disintegrates (no more plasma in the lumen). After the cell death and autolysis, lignin precursors (monolignols) cannot be synthetized any more, however their polymerization to form a lignin macromolecule within the cell wall may still continue (as you describe in lines 384-385 ... After cell autolysis, lignification could continue through releases of vacuolar mognolinol glucosides into the cell wall (Samuels et al., 2002; Pesquet et al., 2013).

We observed a limited degree of post-morten lignification in CML and S1, supporting the idea that it is restricted to outer secondary cell wall layers.

"Abies alba L." is not correct- please change the name to "Abies alba Mill." (everywhere)

Corrected.

Comments to Figure 2.

Fig. 2 a, b please mark where the cambium is Abies alba on Fig. 2c contains traumatic resin canals (TRC). TRC occurrence is due to mechanical injury. This is an exceptional event, therefore TRC are not a part of normal tree ring formation. Lignification of their epithelial cells has another dynamics than normal xylogenesis.

Figure 2 has been modified as suggested, marking the position of the cambial zone in A and B, while indicating the traumatic origin of resin ducts in Abies alba.

Comments to Figure 5: orientation and clarity what is what

The stages of differentiation are now identified in panels A and C, so that regions described in the caption can be easily recognized. The general appearance was also improved by slightly reducing panel dimensions.

Comments to Figure 6: It is difficult to understand this caption. Can you improve it?
The caption has been improved by mentioning panels in the correct order, and by avoiding excessive details on the sampled locations (which resulted confusing). As explained in M&M, Raman profiles were obtained from cell corner and compound middle lamella on nine locations within differentiating xylem. These locations are already shown in Figure 5. For higher detail, their boundaries are shown below.

Raman microscopy mapping of lignin at 1592 to 1604 cm^{-1} (excitation laser: 532 nm) with sampled locations in red.

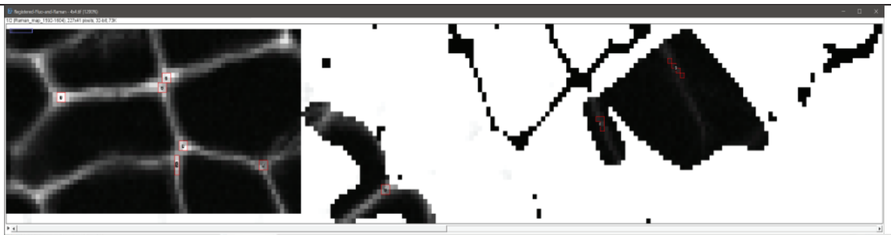

Confocal microscopy mapping of fluorescence emitted at 468 nm (excitation laser: 405 nm) with sampled locations in red

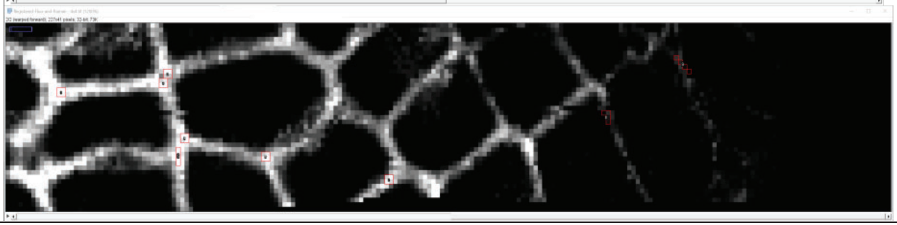

Lines 300-315, please check the wording- it should be precise and careful there is synchronity among wall thickening and lignification- but the lignification is more or less delayed and ends later than the thickening.
We modified the sentence as follows: "…revealing a correspondence between the onset of CML lignification and SCW deposition". We performed some other additional changes in the paragraph to clarify this point.

__**Minor comments (from annotated pdf):**__

Line 15: The dynamics of tracheid lignification are" is grammatically correct.

Line 18: Abies alba L. was replaced by Abies alba Mill. throughout the manuscript.

Line 50: better: wall thickening and lignification, cell maturation, and...
Although cell maturation has been previously used to refer post-enlargement processes (e.g. Wilson et al. 1966), we recognize that it could be confused with "full cell maturation". We thus decided to remove "cell maturation" from the revised version, while including the term "wall thickening" to refer to "SCW deposition + lignification".

Line 56: is the process concurrent with cell wall deposition but slightly delayed
We don't really think there is any conflict between the reviewer statement and the idea that "lignification is the final step of SCW formation" (Meents et al. 2018). Yet, we consider that the information was redundant, so we decided to remove it.

Line 62: Modified by “more recent”.

Line 63: Suggested references included.

Line 64-66: This should be rephrased. In Abies alba tracheids lignification initiates after the polysaccharide matrix starts to be deposited
This paragraph has been improved as suggested. Please, see our reply to general comments.

Line 72: as mentioned in my general comments there is an agreement that name of the phase "W -wall thickening" includes cell wall deposition and lignification
We acknowledge that “cell maturation” is not as widely used as wall thickening, being somehow confusing in relation to full cell maturation. Therefore, we agree that using “wall thickening” to refer to “SCW deposition + cell wall lignification” would be more convenient. This is now clarified in lines 51 and 109 of the Introduction, 231 of M&M and 490 of the Conclusions. To avoid any possible ambiguity, we also removed “cell maturation” from the manuscript (please, see our reply to minor comment in line 50), and replaced “wall thickening” by “SCW deposition” when meaning “cellulose deposition”. In this regard, in the title we replaced “are wall thickening and lignification coordinated?” by “Are cellulose and lignin deposition coordinated?”. Finally, annotations in Figure 1 were modified, including “Wall thickening” in panel A and “Secondary cell wall deposition” and “Lignification” in panel B. However, we think “differentiating and mature cell counts” is correct in this case (line 75) as it also refers to “cell enlargement”.

Line 80: “intra-annual dynamics of lignification have” is grammatically correct.

Line 85: lignification, for instance transmission electron microscopy (TEM) (Donaldson 1992, https://brill.com/view/journals/iawa/13/4/article-p381_3.xml
References to the suggested studies (coauthored by Schmitt and Gricar) have been included in the revised version.

Lines 86-89: Maybe: Besides UV-microspectrophotometry (UMSP) (Koch & Kleist, 2001), the confocal laser scanning (CLSM)...is one of the widely used...
A mention to UV microspectrophotometry has also been included along with the reference to the work by Schmitt et al. (2003).

Line 109-111: According to previous studies on Abies alba this 3 hypotheses should be confirmed
This is right. We slightly modified this paragraph to consider “wall-thickening” as preferred terminology for post-enlargement processes. This particularly affects hypotheses II and III, which are clearer and more straightforward now.

Lines 177: typo corrected.

Line 133: “volume” was replaced by “dimensions” in the revised version of the manuscript.

Line 253: Can you be more precise what do you mean under lumen content?
In the revised manuscript, “lumen content” is replaced by “protoplast”, as we refer to the entire living content of the cell. Fluorescence inside cells was diffuse, so it was not generally possible to identify organelles or structures.

Line 279: higher cellulose proportion?
A higher relative signal intensity for the cellulose band revealed a larger amount of cellulose in location 8 than in location 9 (both enlarging cells), which could be related to a higher proportion. However, cellulose proportion in relation to other cell wall components was not analyzed in the enlarging zone. In the revised version, we replaced "cellulose enrichment of the PCW" by "an increased cellulose content in the PCW" to avoid any ambiguity.

Line 297-298: Resin ducts in Abies alba are traumatic and lignification of the walls of the epithelial cells is a story not related to normal xylogenesis.
We rephrased and moved this sentence to the subsection "Characteristic Raman and autofluorescence signals in mature xylem and the vascular cambium". The traumatic origin of resin ducts in fir (as suggested by the reviewer) is now mentioned, while avoiding speculative ideas in the Results section.

Line 302 and 306: with slight delay of lignification
Please, see our reply in general comments. We rephrased this sentence as follows "In all the studied trees, the number of developing birefringent cells (as noted through TLM) matched the number of lignifying cells counted using CLSM images (**Fig. 7**), revealing a correspondence between the onset of CML lignification and SCW deposition".

Line 346: possibly due to low resolution / magnification. All cell wall layers S1, S2, S3 and warty layer can be recognized by TEM (Schmitt et al., 2003, Gricar et al., 2005) and their degrees of lignification were different.
We agree with the reviewer that distinction between S1 and CLM could have been possible by using a higher magnification, which may have been possible by using other methodologies but also CLSM and Raman. However, the time required for image acquisition and processing given the relatively large number of samples analyzed in CLSM prevented us from using larger image resolution. In the revised manuscript version, we mentioned that limited image resolution hampered distinction of S1.

Line 347: Sentence modified as suggested by the reviewer.

Line 387: yes, to the parts of S2 near the lumen, S3 and warty layer (see Schmitt et al, Gricar et al., 2005).
Thank you for the suggestion. The sentence was modified to include the proposed references.

Line 392: remember that the epithelial cells belong to traumatic resin canals, their development is related to wounding not to normal xylogenesis.
As suggested by the reviewer, we removed "and epithelial cells" from the new version to avoid considerations on anomalous growth responses.

Line 400: this is also related to thicker cell walls in the latewood. See also Prislan et al. 2009
Agree. The sentence was corrected as follows: "Variable lignification periods throughout the tree ring may arise due to an increment in cell wall thickness from early to latewood, but also to seasonal fluctuations in lignin deposition rates".

Line 405: see also Schmitt et al. 2003 and Gricar et al. 2005,
The studies performed by Schmitt et al. (2003), Gricar et al. (2005) are now cited in this paragraph.

Line 422: again Schmitt, Gricar, Prislan.

References included.

Line 423: starts before
We agree, indeed. The sentence was modified as suggested.

Line 424: this depends on time of cell formation i the season and part of the cell wall.
We modified sentence as follows: This cautioned against solely relying on qualitative observations to determine <u>seasonal changes in the degree of overlap</u> between wall thickening and lignification.

Line 434: and in Abies alba
Although there are several lines of evidence supporting a short time lag between SCW deposition and lignification, the linear relationship between cell wall thickness and lignin fluorescence shown by Nanayakkara and colleagues was not shown in other studies (in our opinion). We corrected this sentence to avoid any confusion.

Line 450: but delayed
In the revised manuscript, we added "with a limited amount of lignin being deposited after the end of SCW deposition". In any case, this does not conflict with the idea that most cellulose and lignin in the cell wall are deposited over comparable time windows.

Line 452: Reference included.

Line 464: sorry, this is not the first study
Likely, "cell-based approach" was not an accurate term in this context. This sentence has been rephrased to better describe the novelty of our study, which is the first (to the best of our knowledge) providing an estimation of timings and durations of both wall thickening and lignification along the tree ring.

Line 493: I am not shure for this statement
For higher accuracy, we reorganized ideas and modified this sentence as follows: "these results suggest that most cellulose and lignin are deposited in the xylem over similar timeframes".

Line 495: Modified as suggested.

Line 503: Replaced by "our study offers new perspectives…"

Line 505: Modified as suggested.

18-Mar-2024
Dr. Gonzalo Pérez-de-Lis
Universidade de Santiago de Compostela
Lugo
Spain

MSID: PP2023-RA-01883R1
MS TITLE: Are cellulose and lignin deposition coordinated? A multimodal imaging analysis of xylogenesis in silver fir

Dear Dr. Gonzalo Pérez-de-Lis:

I am pleased to inform you that your revised manuscript "Are cellulose and lignin deposition coordinated? A multimodal imaging analysis of xylogenesis in silver fir" has been accepted for publication in Plant Physiology. Your manuscript will be checked for consistency with journal formatting and image standards by one of our Science Editors, who will contact you shortly. They may have a number of queries and suggestions for you. Please keep in mind that they work to increase the online visibility of your article and its attraction for readers from the widest of backgrounds. You will have the opportunity to review any revisions, and I hope you will work constructively with them to get the most out of your research. You will also have the opportunity to review all changes when you receive the author proofs for your article.

Once validated for publication, your article will be sent to a Science Editor for a final review of your manuscript and source files. After your final files are uploaded, your article will appear in the next available issue and the online Preview version of your article will be posted after your signed Copyright and Checklist forms and final source files are received.

Please be sure to include the following in your manuscript file:
Harvard style citations
List of all author contributions and funding information at the beginning of manuscript after the titles and author list.
List all supplemental materials associated with the article, with titles, at the end of the ms, before the references and acknowledgments section.
Complete Figure Legends for all figures appearing in the article listed before the Literature Cited.

Thank you and congratulations on your Plant Physiology paper!

Sincerely,
Board of Editors Plant Physiology

=====
FIRST AUTHOR PROFILES
=====

We recognize the hard work that goes into being First Author of a Plant Physiology paper by publishing a "First Author Feature" for each First Author alongside your paper.

If the first author(s) would like to submit information for a feature on Facebook and Twitter, fill out the form by clicking on the link below:

Plant Physiology First Author Feature Form: https://forms.office.com/r/ZBCGZfhm8F
If you cannot access the form through the link above, please contact Rachel at rbelsky@aspb.org.

To upload an image of yourself to accompany your profile, please put your photo in the correct journal folder at the link below. You must label your image: Author First Name Last Name Article Title.

First Author Profile Image Dropbox: https://www.dropbox.com/scl/fo/xz029p7qjr2jml2rp2kmq/h?dl=0&rlkey=6fkxcaqmxx35ap152b2osy1fz

=====
IMPORTANT REMINDER: PEER REVIEW REPORTS

=====

If you opted to publish a peer review report along with your article during the original submission process, it will be prepared by the editorial staff and publicly posted with your manuscript, inside the zip file that contains any other supplemental material. As a reminder, the peer review report is a public record of all comments from editors and reviewers, as well as your prior responses, as you received them in the decision letters for each draft of your manuscript. If you agreed to publish this report and have changed your mind, or are not sure if you selected this option, please contact the editorial office as soon as possible before signing the license agreement from our publisher.

-- Reviewer comments:
